# Ezh1 arises from Ezh2 gene duplication but its function is not required for zebrafish development

**DOI:** 10.1038/s41598-019-40738-9

**Published:** 2019-03-13

**Authors:** Pamela Völkel, Aurélie Bary, Ludivine Raby, Anaïs Chapart, Barbara Dupret, Xuefen Le Bourhis, Pierre-Olivier Angrand

**Affiliations:** 1 Inserm U908, Cell Plasticity & Cancer, Lille, France; 2 University of Lille, Lille, France; 3 CNRS, Lille, France; 4 FRABio, CNRS FR3688, Lille, France

## Abstract

Trimethylation on H3K27 mediated by Polycomb Repressive Complex 2 (PRC2) is required to control gene repression programs involved in development, regulation of tissue homeostasis or maintenance and lineage specification of stem cells. In *Drosophila*, the PRC2 catalytic subunit is the single protein E(z), while in mammals this function is fulfilled by two proteins, Ezh1 and Ezh2. Based on database searches, we propose that *Ezh1* arose from an *Ezh2* gene duplication that has occurred in the common ancestor to elasmobranchs and bony vertebrates. Expression studies in zebrafish using *in situ* hybridization and RT-PCR followed by the sequencing of the amplicon revealed that *ezh1* mRNAs are maternally deposited. Then, *ezh1* transcripts are ubiquitously distributed in the entire embryo at 24 hpf and become more restricted to anterior part of the embryo at later developmental stages. To unveil the function of *ezh1* in zebrafish, a mutant line was generated using the TALEN technology. Ezh1-deficient mutant fish are viable and fertile, but the loss of *ezh1* function is responsible for the earlier death of *ezh2* mutant larvae indicating that *ezh1* contributes to zebrafish development in absence of zygotic *ezh2* gene function. Furthermore, we show that presence of *ezh1* transcripts from the maternal origin accounts for the delayed lethality of *ezh2*-deficient larvae.

## Introduction

Polycomb group (PcG) proteins are epigenetic regulators conserved from fruit flies to humans. They are involved in various biological processes including regulation of tissue homeostasis, maintenance and lineage specification of stem cells, and promote cancer progression when skewed^1–4^. PcG proteins assemble in two main histone-modifying protein complexes named Polycomb Repressive Complexes 1 and 2 (PRC1 and PRC2). PRC2 catalyzes the methylation of Lysine 27 of histone H3, generating the H3K27me2/3 epigenetic mark which in turn acts as a platform to recruit the PRC1 complex that ubiquitylates histone H2A at Lysine 119 (H2AK119ub1)^5–10^. These post-translational modifications are then responsible for local chromatin compaction and gene silencing.

The PRC2 protein complex is composed of several subunits but its core comprises Enhancer of zeste homolog (Ezh), Embryonic ectoderm development (Eed) and Suppressor of zeste 12 (Suz12)^2^. *Drosophila* have a single Ezh gene [E(z)], whereas mammalian genomes encode two orthologs^11^ defining two alternate PRC2 complexes, PRC2-Ezh1 and PRC2-Ezh2. Ezh1 and Ezh2 are SET domain-containing proteins harboring the histone methyltransferase activity, while Eed and Suz12 are involved in PRC2 stability and are required for Ezh1/2 catalytic activities^12–16^. Although Ezh2 knockout in embryonic stem cells (ESCs) strongly reduces H3K27me2/3, H3K27 methylation is not fully abolished suggesting that PRC2-Ezh1 complexes contribute to this epigenetic mark formation^17^. In contrast to PRC2-Ezh2, PRC2-Ezh1 exhibits a low histone methyltransferase activity and knockdown of Ezh1 does not result in global reduction of H3K27me2/3 levels^13^. However, PRC2-Ezh1 represses transcription *in vivo* and is able to compact chromatin *in vitro*^13^. Also, depletion of Ezh1 in cells lacking Ezh2 abolishes residual methylation on H3K27^17^. In addition, *Ezh1* is ubiquitously expressed, whereas *Ezh2* expression is associated with proliferating tissues^13^. Finally, *EZH2* has been largely documented to be involved in tumorigenesis^18^, which it is also the case for *EZH1*. In particular, mutations in EZH1 on Glutamine 571 (Q571R) were found to occur in more than 25% of adult autonomous thyroid adenomas^19^. This EZH1^Q571R^ mutation lies within the SET domain of EZH1 and is responsible for an increase of H3K27me3 methylation.

The zebrafish (*Danio rerio*) provides a unique tool to investigate gene function during development and provides important models for human diseases. Owing to external fertilization and optical transparency of the embryos, zebrafish early development can be easily monitored. Furthermore, the recent emergence of powerful genome-editing technologies, such as the Transcription Activator-Like Effector Nuclease (TALEN) and Clustered Regularly Interspaced Short Palindromic Repeats/CRISPR-associated System (CRISPR/Cas9) applied to zebrafish allows rapid gene function studies in this organism^20–25^. We previously used the TALEN-mediated gene editing methodology to generate an *ezh2*^*ul2*^ null allele in zebrafish^26^. Mouse embryos lacking *Ezh2* function fail to complete gastrulation^27^, but *ezh2*^*ul2/ul2*^ zebrafish mutants present a normal body plan and die at around 12 days post fertilization (dpf) with intestinal defects. In contrast, maternal-zygotic (MZ) *ezh2* zebrafish mutants generated through germ cell transplantation (*MZezh2*^*hu5670/hu5670* ^^28^) also gastrulate and form a normal body organization, but die at around 2 dpf. The difference in the lethality timing between zygotic and maternal-zygotic *ezh2* mutants outlines the key role played by *ezh2* maternal products in zebrafish development. However, the fact that *MZezh2*^*hu5670/hu5670*^ mutants develop a normal body plan raises the possibility that *ezh1* could contribute to early embryogenesis in absence of *ezh2* function.

Using searches in vertebrate genomic and transcriptomic databases, we first show here that *ezh1* arises from the duplication of the *ezh2* gene in the common ancestor to elasmobranchs and bony vertebrates. On the other hand, we conducted a study of *ezh1* expression and function during zebrafish development. Using the TALEN technology, we generated an *ezh1* loss-of-function zebrafish line. This line is viable and fertile indicating that Ezh1 is dispensable in zebrafish. However, we show that *ezh1* contributes to zebrafish development in absence of zygotic *ezh2* gene function.

## Results

### Phylogenetic analysis of Ezh1

**Figure 1:**
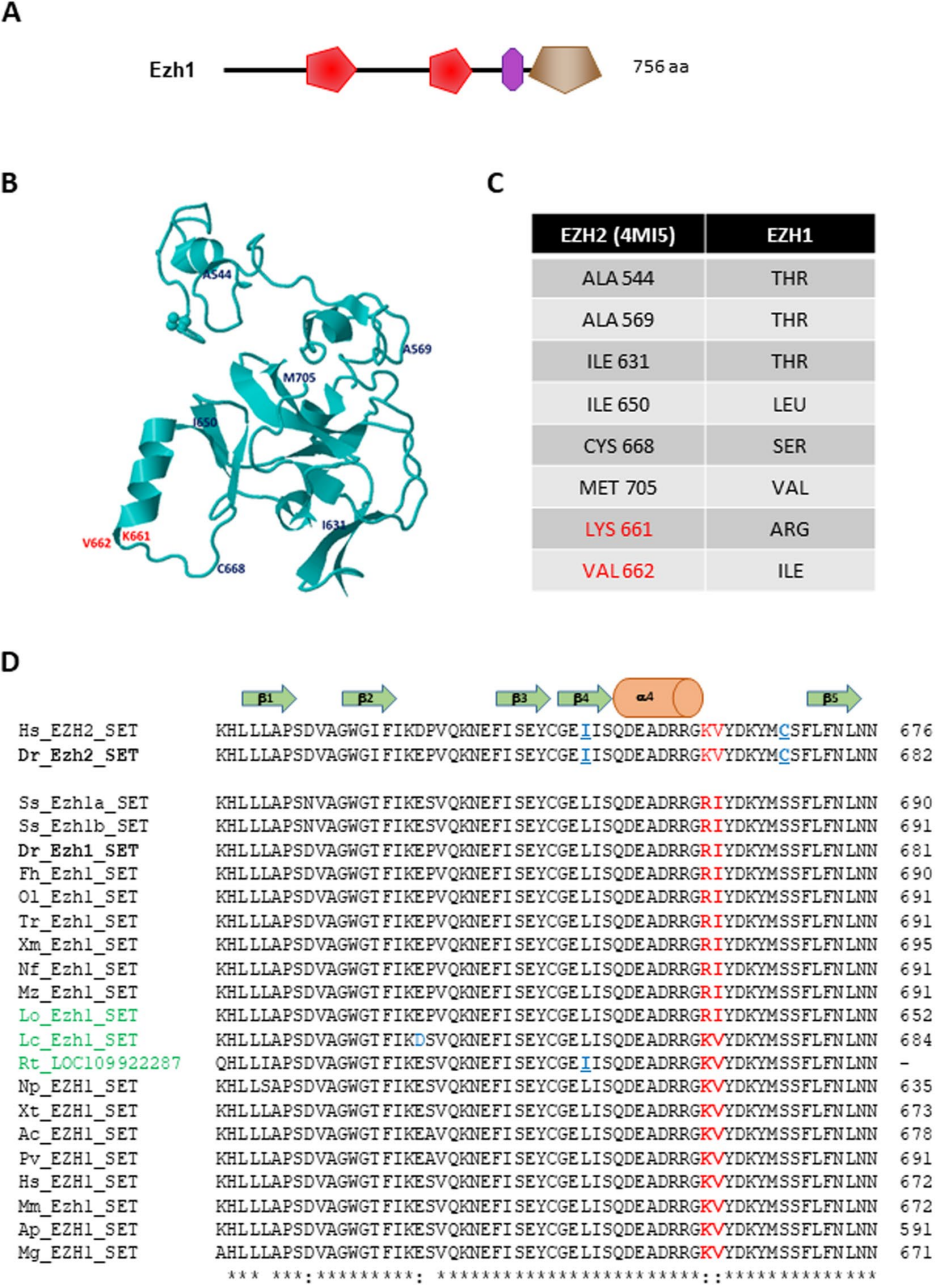
Structure of Ezh1 and its SET domain. (**A**) Schematic representation of the zebrafish Ezh1 protein. Red, violet and brown motifs correspond to SANT (SMART: SM00717), CXC (SMART: SM01114) and SET (SMART: SM00317) domains, respectively. The size of the protein is indicated. (**B**) 3D-structure of the human EZH2 SET domain (PDB ID: 4MI5) represented as a ribbon model^30^. The position of the substitutions identified in Ezh1 proteins are shown. Substitutions specific to ray-finned fish are in red. (**C**) List and nature of the substitutions identified in Ezh1 proteins are shown. Substitutions specific to ray-finned fish are in red. (**D**) Multiple protein alignment of the region of the SET domains between the β-sheets β1 and β5 from human (Hs) and mouse (Mm) Ezh2 and Ezh1 from several vertebrates. Hs: *Homo sapiens*; Mm: *Mus musculus*; Ss: *Salmo salar*; Dr: *Danio rerio*; Fh: *Fundulus heteroclitus*; Ol: *Oryzia latipes*; Tr: *Takifugu rubripes*; Xm: *Xiphophorus maculatus*; Nf: *Nothobranchius furzeri*; Mz: *Maylandia zebra*; Lo: *Lepisosteus oculatus*; Lc: *Latimeria chalumnae*; Rt: *Rhincodon typus*; Np: *Nanorana parkeri*; Xt: *Xenopus tropicalis*; Ac: *Anolis carolinensis*; Pv: *Pagona vitticeps*; Ap: *Anas platyrhynchos*; Mg: *Meleagris gallopavo*. The residues Ile650 and Cys668 are shown in blue and underlined in the EZH2 SET sequence, while Lys661 and Val662 are in red. Non-teleostean fish are indicated in green.

Amongst the about 50 histone lysine methyltransferases encoded by the mammalian genomes, Ezh1 and Ezh2 are the only enzymes able to perform the H3K27me2/3 methylation. Furthermore, Ezh1 and Ezh2 present a unique protein architecture composed of 2 SANT domains (SMART ID: SM00717), a catalytic SET domain (SMART ID: SM00317) and a pre-SET domain rich in Cysteines, N-terminal to the SET domain and comprising a CXC domain (SMART ID: SM01114) (Fig. 1A)^11,29^. The 3D-structure of the catalytic domain of human EZH2^30,31^ reveals that the pre-SET domain is organized as two three-atom clusters of bound zinc coordinated by two distinct nine-residues. The first three zinc atoms are coordinated by Cysteines 528, 535, 539, 541, 548, 552, 554, and 558 together with Histidine 530. The second group of three zinc atoms is coordinated by Cysteines 565, 567, 571, 576, 578, 585, 590, 593, and 606. Each zinc binding domain contains a short helical structure (α1 and α2) which is formed just after the fourth zinc binding cysteine found in the cluster. The core of the SET domain is formed by 2 three-stranded anti-parallel β-sheets (β3, β7, β8) and (β4, β5, β6) diagonally pressed across each other and flanked by 2 short α-helices α4 and α5 (Fig. 1B)^30,31^.

Sequence comparison between the SET domains of Ezh1 and Ezh2 from two mammals, the human (*Homo sapiens*) and the mouse (*Mus musculus*), two birds, the mallard (*Anas platyrhynchos*) and the turkey (*Meleagris gallopavo*), two reptiles, the green anole (*Anolis corolinensis*) and the bearded dragon (*Pogona vitticeps*), two amphibians, the western clawed frog (*Xenopus tropicalis*) and the high Himalaya frog (*Nanorana parkeri*) and two fishes, the zebrafish (*Danio rerio*) and the medaka (*Oryzia latipes*) reveals a limited number of amino-acid substitutions specifying the two different catalytic domains (Supplementary Fig. S1). These substitutions, A544T, A569T, I631T, I650L, C668S and M705V are located in different regions of the SET domain (Fig. 1B,C) and will be used to distinguish Ezh1 from Ezh2 in Blast searches in databases. Furthermore, we identify two additional amino-acid substitutions specific to zebrafish and medaka Ezh1 SET domains after α4, the changes K661R and V662I (Fig. 1B,C and Supplementary Fig. S1).

To identify Ezh1 from teleost genomes, the sequences of the SET domains from human and zebrafish Ezh1 proteins were used in independent TBLASTN searches against genome databases (NCBI, https://blast.ncbi.nlm.nih.gov/Blast.cgi; Ensembl, http://www.ensembl.org/Multi/Tools/Blast?db = core; EFish Genomics, https://efishgenomics.integrativebiology.msu.edu/blast_search/) for 25 teleost species (Supplementary Table S1). At least one gene encoding Ezh1 could be identified in each teleostean checked (Supplementary Table S1). Two ezh1 genes (LOC108928200 and LOC108941744) are found in the Asian arowana (Scleropages formo*sus*) genome. This is due to the fact that a whole-genome duplication, termed the teleost-specific third whole-genome duplication (Ts3R) has occurred in the teleost lineage after it splits from the tetrapods^32,33^. Following whole-genome duplication, the resulting duplicated genomes eventually retain only a small part of the duplicated genes (onhologs). Thus, while the Asian arowana possesses two duplicated *ezh1* genes, most of the other teleosts retain only one gene. The finding that the Asian arowana has two *ezh1* onhologs parallels the fact that this fish genome contains a complete set of post-Ts3R complement for different genes including eight Hox clusters, while other teleost genomes retain less Hox clusters [7 in the zebrafish and even 5 in the African butterfly fish (*Pantodon buchholzi*)]^34^. The common carp (*Cyprinus carpio*) also contains two *ezh1* onhologs (LOC109102790 and LOC109057529), but arising from a more recent whole-genome duplication since the carp is a tetraploid fish containing about 100 chromosomes, approximately twice the number of most other cyprinidae, including zebrafish^35,36^. Finally, in agreement with a relatively recent whole-genome duplication event, the salmonid-specific fourth whole-genome duplication (Ss4R)^37^ having occurred in the salmonidae family of teleosts fishes, the Atlantic salmon (*Salmo salar*), the rainbow trout (*Oncorhynchus mykiss*) and the Coho salmon (*Oncorhynchus kisutch*) possess two *ezh1* copies in their haploid genomes (Supplementary Table S1). The analysis of the Ezh1 SET domain sequences in teleost fishes reveals that the K661R and V662I substitutions are present in all teleostean Ezh1 proteins suggesting that these changes occurred before the Ts3R genomic event (Fig. 1D). Holostei are composed of eight living species of ray-finned fish (Actinopterygii) whose lineage diverged from teleosts before the Ts3R genome duplication. Among them, the genome of the spotted gar (*Lepisosteus oculatus*) has been sequenced^38^. The genome of the spotted gar contains one *ezh1* gene (NCBI Gene ID: 102693111, Supplementary Table S1) coding for a protein having a SET domain also harboring the K661R and V662I substitutions (Fig. 1D). In contrast, these two amino-acid changes were not found in the Ezh1 SET domain of the African coelacanth (*Latimeria chalumnae*) which belongs to the class of lobe-finned fish (Sarcopterygii) (Fig. 1D)^39^. Then, the two amino-acids changes at K661R and V662I are not specific to the teleosts. They are found in other ray-finned fish including the holosteian spotted gar, but not in the lobe-finned fish nor in the other tetrapods.

We next searched for the presence of the *ezh1* gene in the cartilaginous fish genomes. Cartilaginous fishes (Chondrichthyes) comprise two subclasses, the elasmobranchs including the sharks, the rays, the skates and sawfish, and the holocephalans composed of several chimera species, thought to have diverged from a common ancestor more than 400 million years ago (Supplementary Fig. S2). Using human and zebrafish Ezh1 sequences in TBLASN searches, we identified two genomic sequences in the whale shark (*Rhincodon typus*) genome^40^ (Supplementary Table S1). LOC109924829, located at one extremity of the genomic clone NW_018048782.1, codes for a partial protein containing 2 SANT domains and a part of a CXC domain with high homology to Ezh1, whereas LOC109922287, within the genomic clone NW_018043781.1, potentially contains the last 5 exons of a gene coding for an Ezh1 SET domain (Supplementary Fig. S3). It is then likely that LOC109924829 and LOC109922287 correspond to two parts of the whale shark *ezh1* gene. Alignment of the whale shark Ezh1 SET domain with other Ezh1 and Ezh2 SET domains reveals that the Ezh1 SET domain from the whale shark does not contain the K661R and V662I substitutions specific to the Ezh1 SET domains of the ray-finned fish. However, it possesses the Ezh1-specific substitutions I631T, C668S and M705V, but not I650L (Supplementary Fig. S3 and 1D). The little skate (*Leucoraja erinacea*) is the second elasmobranch for which genomic and transcriptomic sequences are available in databases^41,42^. Using human and zebrafish Ezh1 sequences in a TBLASN search in the Skatebase (http://skatebase.org), we identified a partial transcript from the little skate (LS-transcriptB2-ctg80080) that codes for a SET domain highly related to Ezh1. As for the whale shark, the Ezh1 SET domain of the little skate possesses the Ezh1-specific substitutions C668S and M705V, but not I650L nor the ray-finned fish-specific K661R and V662I changes (Supplementary Fig. S3).

In contrast, we failed to detect *ezh1* in a jawless vertebrate, the sea lamprey (*Petromyzon marinus*) while the *ezh2* gene could be found (ENSPMAG00000004401), suggesting that ezh2 is more ancient and that *ezh1* might have arisen from the duplication of *ezh2*.

**Figure 2:**
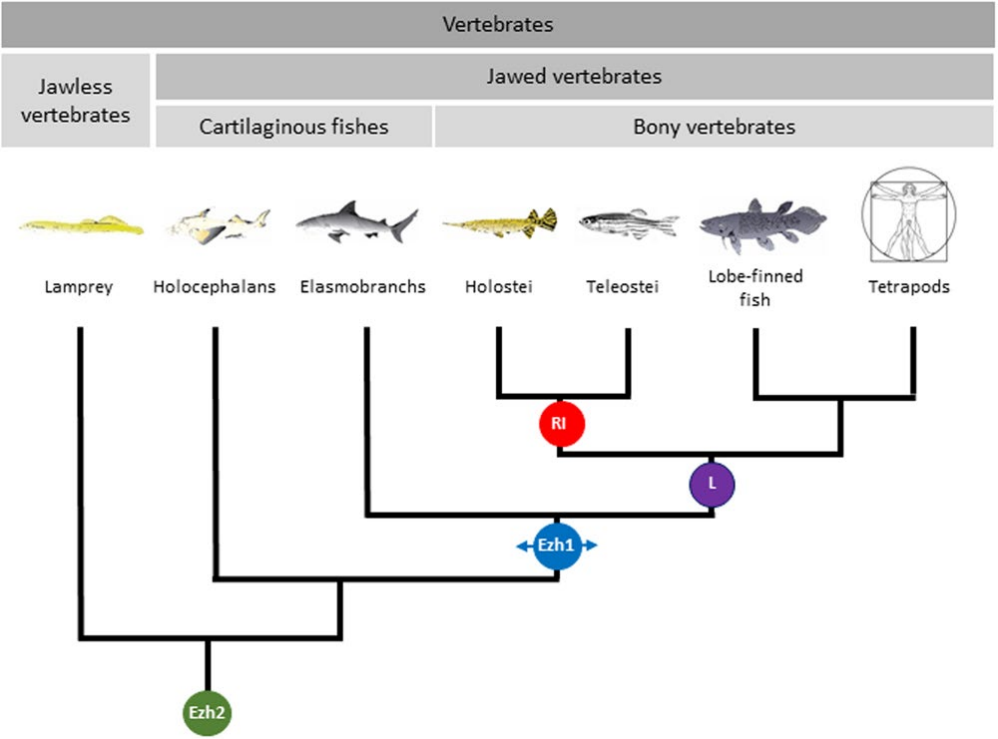
Proposed phylogeny of vertebrates based on *Ezh1-Ezh2* evolution. Proposed phylogenic tree showing changes that have occurred in Ezh1/2 SET domains during evolution. Ancient vertebrates contain only Ezh2 in their genome (Green circle). *Ezh1* arose from *Ezh2* gene duplication in the common ancestor to elasmobranchs and bony vertebrates (Blue circle). Additional substitutions such as the SET I650L change occurred in the common ancestor to bony vertebrates (Violet circle). Ray-finned fish including at least teleostans and holosteans harbor specific Ezh1 SET domain substitution such as K661R and V662I (Red circle). The drawings were done by Shaghayegh Hasanpour.

The elephant shark (*Callorhinchus milii*) is the only holocephalan having its genome sequenced^43^. Using TBLASTN searches, we also failed to identify an *ezh1* gene in the elephant shark genome while *ezh2* could be found (NCBI Gene ID: 103187005). This suggests that the *ezh2* duplication might have occurred after the holocephalans and elasmobranchs have diverged. It also implies that there is an ancestor common to elasmobranchs and to bony vertebrates, but not to holocephalans in which this *ezh2* duplication happened (Fig. 2).

Consequently, we propose that like in *Drosophila* and other invertebrates that have a single *ezh* gene [*E(z)*], the first vertebrates possessed only *ezh2* in their genomes. That is still the case for lampreys and chimeras (Fig. 2). Ezh1 harboring several amino-acid substitutions such as C668S and M705V in the SET domain, appeared in the common ancestor of elasmobranchs and bony vertebrates following an *ezh2* gene duplication. In the common ancestor of ray-finned fish, lobe-finned fish and tetrapods additional changes like the SET domain I650L substitution, were introduced in Ezh1. Finally, ray-finned fish including teleosteans and holosteans contain other substitutions including the SET domain K661R and V662I changes (Fig. 2).

### Ezh1 expression during zebrafish development

**Figure 3:**
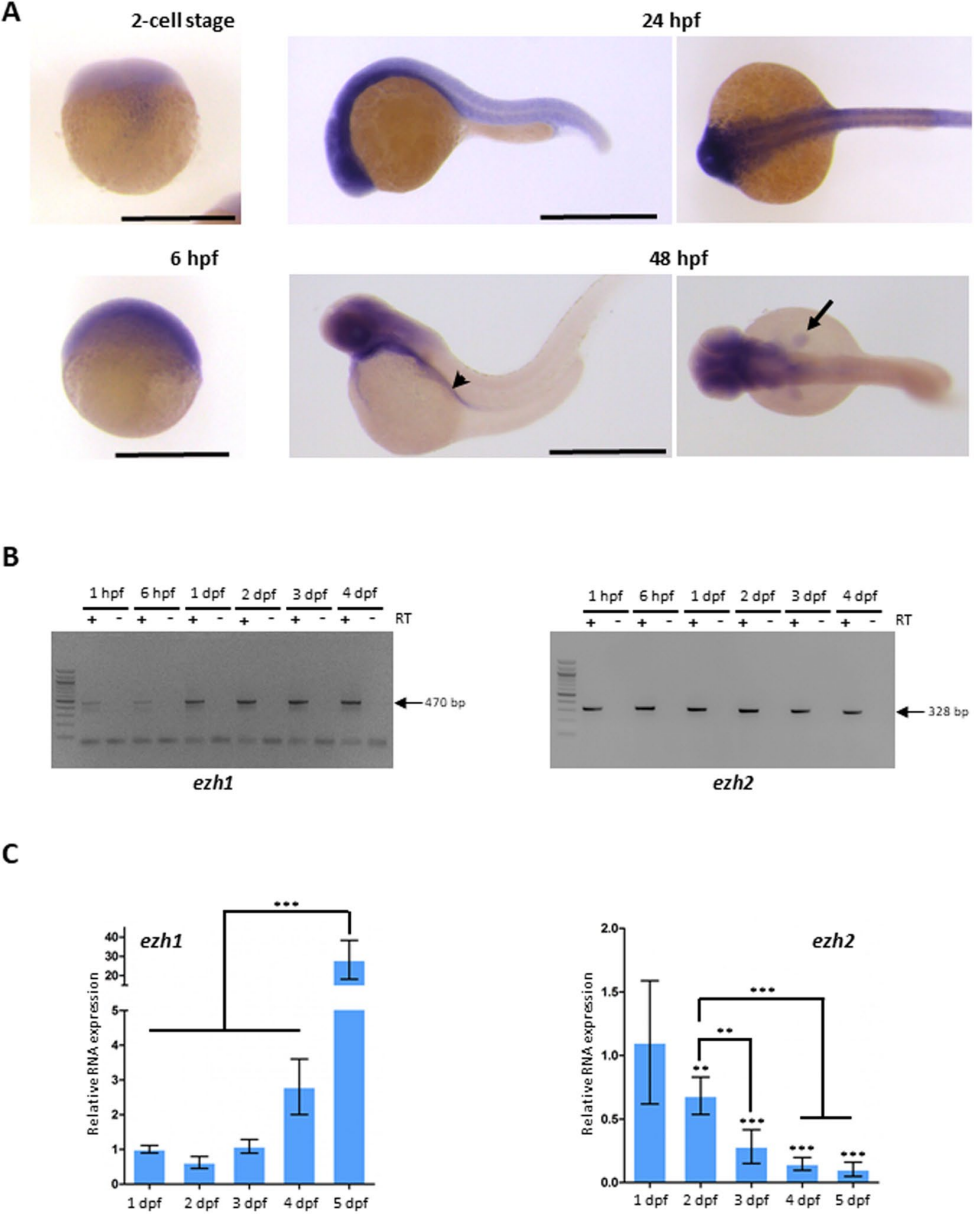
*Ezh1* expression during zebrafish development. (**A**) *In situ* hybridization at the 2-cell stage, 6 hpf, 24 hpf and 48 hpf showing that *ezh1* transcripts are maternally provided (2-cell stage) and showing zygotic *ezh1* mRNA distribution during early development. The arrowhead shows the gut and the arrow the pectoral fin bud. Scale bar is 500 μm. (**B**) RT-PCR analysis showing detection of *ezh1* and *ezh2* mRNAs at 1 hpf, 6 hpf, 1 dpf, 2 dpf, 3 dpf and 4 dpf. The reverse transcriptase (RT) was included (+) or not (−) in the reaction, as indicated. (**C**) RT-qPCR showing that relative *ezh1* expression increases during larval development from 1 dpf to 5 dpf whereas *ezh2* expression decreases. Three independent experiments were performed and error bars represent standard deviation. Statistical analysis was performed using a one-way ANOVA with Tukey’s *post hoc* test. **P < 0.01; ***P < 0.001.

In zebrafish, zygotic transcription starts at the midblastula transition at about cell cycle 10–13 [3–4 hours post fertilization (hpf)]. Before this stage, all developmental processes depend on maternally deposited gene products^44,45^. We studied the expression pattern of *ezh1* before the midblastula transition (2-cell stage), as well as at later developmental stages (6, 24 and 48 hpf). Whole-mount *in situ* hybridization reveals that the *ezh1* transcript is maternally loaded into the embryos since a signal could be detected before midblastula transition at the 2-cell stage (Fig. 3A). To demonstrate that the hybridization conditions are stringent enough and that the *ezh1* antisense probe used in the experiments is specific to *ezh1* transcripts and does not hybridize with mRNAs encoding other SET domain-containing histone methyltransferases including *ezh2*, we performed whole-mount *in situ* hybridization on wild-type and maternal-zygotic *MZezh1*^*ul3/ul3*^ mutant (see below) embryos at the 1 to 2-cell stages (1 hpf) (Supplementary Fig. S4). A clear *ezh1* signal is detected in the wild-type embryos whereas the signal is strongly reduced in *MZezh1*^*ul3/ul3*^ mutant embryos and absent when an *ezh1* sense probe is used in the experiments, thus demonstrating the specificity of the *ezh1* RNA probe (Supplementary Fig. S4). The reduction of the *ezh1* signal in *MZezh1*^*ul3/ul3*^ mutant embryos probably relies on a non-sense mediated decay mechanism which appears to be less intense at later developmental stages, as observed for *ezh2* in zygotic *Zezh2*^*ul2/ul2*^ mutants^26^. The presence of *ezh1* mRNAs in embryos before the midblastula transition is further confirmed by RT-PCR (Supplementary Fig. S5). The PCR primers used in the RT-PCR experiments amplify the *ezh1* transcript (*ezh1-001*, ENSDART00000039170.8) as well as a predicted shorter transcript (*ezh1-002*, ENSDART00000101965.6) potentially coding for an Ezh1 isoform lacking the SANT and SET domains. However, these two transcripts could be distinguished by RT-PCR according to their size since the intron 4 is retained in the short transcript, but not in *ezh1* (*ezh1-001*) mRNA. Then, RT-PCR amplification is expected to give 470 bp and 871 bp fragments corresponding to the *ezh1* (*ezh1-001*) transcript and the predicted shorter *ezh1-002* transcript, respectively. RT-PCR experiments performed on mRNAs extracted from 1 hpf embryos identify an amplified DNA fragment at about 470 bp, but not at 871 bp suggesting that *ezh1* (*ezh1-001*) transcripts, but not the predicted shorted *ezh1-002* transcripts are maternally loaded in zebrafish embryos. The sequencing of the amplicon confirms that it corresponds to *ezh1-001*, but not to *ezh2*, nor to *ezh1-002* as the intron 4 is spliced out (Fig. 3B, Supplementary Fig. S5). Altogether, our results based on both *in situ* hybridization and RT-PCR followed by sequencing, demonstrate that *ezh1* transcripts are maternally deposited into the zebrafish embryos.

At later developmental stages, zygotic *ezh1* expression is detected at the 50%-epiboly stage (6 hpf) (Fig. 3A). At 24 hpf, *ezh1* mRNAs are ubiquitously present in the embryo and expression becomes more restricted in the developing brain and in the pectoral fin buds at 48 hpf (Fig. 3A). RT-PCR experiments show that *ezh1* RNA levels are higher at 24 hpf compared to 1 or 6 hpf suggesting that maternal levels of *ezh1* transcripts are low (Fig. 3B).

Quantitative RT-qPCR reveals that *ezh1* mRNA levels increase during early larval development between 24 hpf and 5 dpf. This contrasts with *ezh2* mRNA levels which are decreasing during the same larval developmental period (Fig. 3C).

### TALEN-mediated *ezh1* inactivation in zebrafish

**Figure 4:**
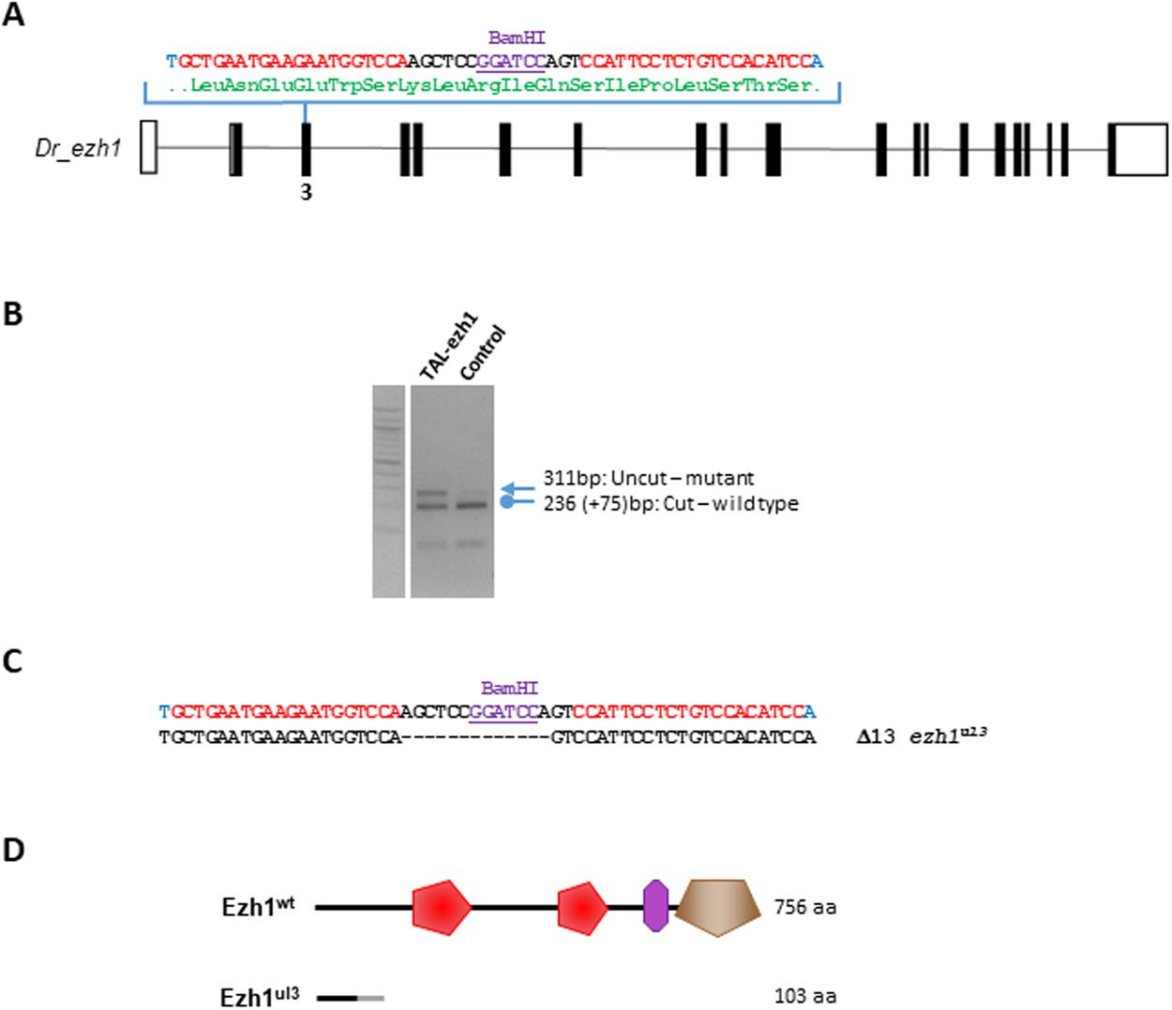
Generation of *ezh1* mutant zebrafish using the TALEN technology. (**A**) Schematic representation of the genomic structure of the *ezh1* gene, with coding and untranslated sequences depicted as solid and open boxes, respectively. The location of the *ezh1* TALEN in exon 3 (3) is indicated. The *ezh1* TALEN target sequence with Left and Right TALEN binding sites in red is shown. The BamHI restriction site is underlined and indicated in violet. (**B**) Identification of mutant embryos using restriction fragment length polymorphism. Genomic DNA was prepared from an uninjected (Control) and an *ezh1* TALEN injected (TAL-ezh1) embryo, amplified by PCR and subjected to BamHI digestion. The TAL-ezh1 injected embryo contains undigested material (arrow at 311 bp), indicating that the BamHI diagnostic restriction site has been disrupted. (**C**) Sequence of the mutant allele compared to its wild-type counterpart. Dashes indicate deleted nucleotides. The mutated *ezh1*^*ul3*^ allele has a deletion of 13 nucleotides. (**D**) Schematic representation of predicted the wild-type (Ezh1^wt^) and mutant (Ezh1^ul3^) proteins. The gray line in the predicted mutant protein indicates residues read out of frame prior to encountering a premature stop codon, whereas the red, violet and brown motifs in the wild-type protein correspond to SANT (SMART: SM00717), CXC (SMART: SM01114) and SET (SMART: SM00317) domains, respectively. Sizes of the predicted proteins are indicated.

To gain insights into the function of *ezh1* in zebrafish development, we generated *ezh1* loss-of-function mutants using the transcription activator-like effector nuclease (TALEN)-based technology. TALENs were designed to target the third exon of *ezh1* in order to introduce a frameshift upstream of all known Ezh1 conserved domains, such as the SANT, CXC and SET domains. In addition, the targeted region was chosen to contain a BamHI restriction site used to screen for mutations and for genotyping analyses (Fig. 4A). TALENs were assembled using the Golden Gate Cloning methodology^46^ and *in vitro* transcribed mRNAs encoding each TALEN pair were injected into one-cell stage embryos. At 3 days post injection, genomic DNA was extracted from single embryos and PCR amplification of the targeted region, followed by BamHI digestion revealed the efficacy of the TALENs and of the BamHI restriction strategy for genotyping (Fig. 4B).

**Figure 5:**
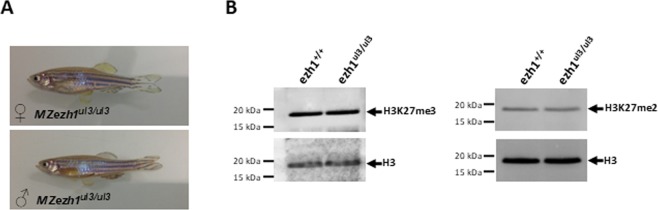
*Ezh1* ul3/ul3 zebrafish mutants present a normal phenotype. (**A**) Pictures of 6 month-old *MZezh1*^*ul3/ul3*^ mutant female (top) and male (bottom) zebrafish from the F2 generation of incross mutant fish. (**B**) Global trimethylation (H3K27me3) and demethylation (H3K27me2) of lysine 27 of histone H3 is not affected in *ezh1*^*ul3/ul3*^ mutants. Total histones from pools of 10 larvae at 9 dpf from wild-type or *ezh1*^*ul3/ul3*^ crosses as indicated, were extracted and 5 µg of total histones were analyzed by western blotting using specific anti-H3K27me3 or anti-H3K27Me2 antibodies and an anti-H3 antibody as a control.

Restriction analysis of genomic DNA showed that the mutation rate at the *ezh1* locus was 100% (29 out of 29 injected embryos tested). Then, we raised TALENs-injected embryos to establish an adult F0 founder population. To evaluate the efficiency of germ line transmission of the mutations, individual F0 fish carrying *ezh1* mutations (loss of the BamHI restriction site) were outcrossed to wild-type TU to obtain F1 offspring. Genomic DNA was isolated from individual F1 embryos from each F0 fish and analyzed by BamHI restriction. Embryos from 3 of 8 individual F0 fish were heterozygous mutants, demonstrating successful germ line transmission of the mutations. Among them, one mutation causes a 13 bp deletion leading to a frame shifting of the coding sequence and appearance of a premature stop codon (Fig. 4C). This Δ13 *ezh1* allele, hereafter called *ezh1*^*ul3*^, codes for a predicted protein of 103 amino acids, lacking all conserved protein domains (Fig. 4D, Supplementary Fig. S6) and was selected to raise a heterozygous *ezh1*^+*/ul3*^ zebrafish line. The heterozygous *ezh1*^+*/ul3*^ fish are viable, fertile and do not show any phenotype. Among siblings from heterozygous *ezh1*^+*/ul3*^ incrosses, we successfully identified males and females carrying the homozygous Δ13 mutation (*ezh1*^*ul3/ul3*^). The homozygous mutants are viable and fertile allowing us to generate a maternal-zygotic *MZezh1*^*ul3/ul3*^ line used for further phenotypic studies. Fish from this *MZezh1*^*ul3/ul3*^ line are still fertile after at least two incross generations and do not present any phenotype (Fig. 5A) demonstrating that both maternal and zygotic *ezh1* products are dispensable to zebrafish development.

Since Ezh1 is a histone methyltransferase able to promote H3K27me2/3 methylation, we next evaluated the H3K27me2/3 status of the *ezh1*^*ul3/ul3*^ line. Total histones were extracted from mutant and wildtype larvae at 9 dpf and H3K27me3 was analyzed by western blot using specific anti-H3K27me3 and anti-H3K27me2 antibodies. Figure 5B reveals that global levels of H3K27me2/3 are similar in *MZezh1*^*ul3/ul3*^ and wild-type total larvae, indicating that loss of *ezh1* function does not affect global levels of H3K27me2/3 methylation in zebrafish.

### Loss of *ezh1* function leads to an increase in *ezh2* expression

**Figure 6:**
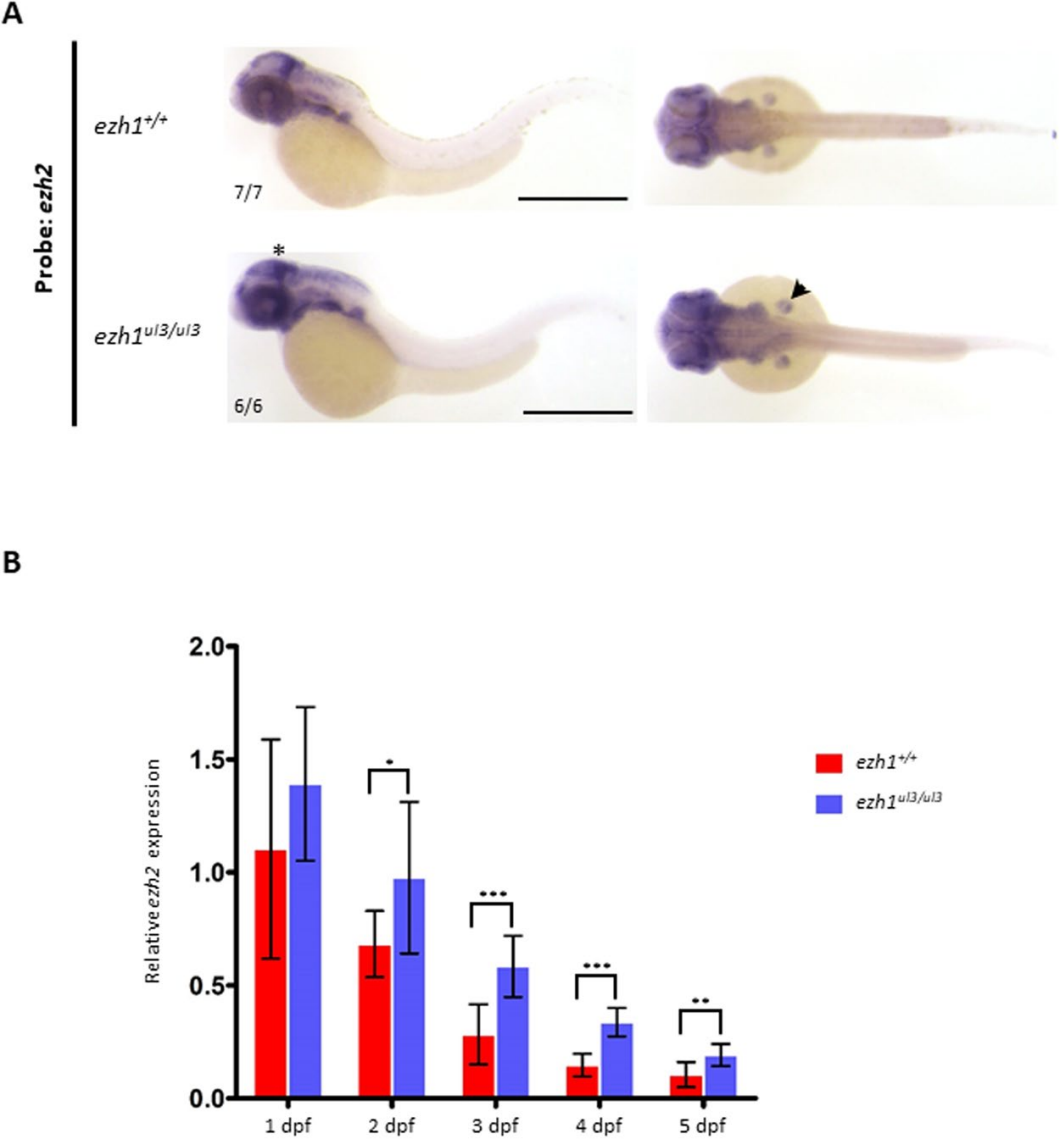
Loss of ezh1 function leads to an increase in ezh2 expression. (**A**) *In situ* hybridization showing *ezh2* expression in representative embryos at 48 hpf from wild-type (Top) and *ezh1*^*ul3/ul3*^ crosses. A slight increase in the *ezh2* signal at both sides of the midbrain-hindbrain boundary (asterisk) and in the pectoral fin buds (arrowhead) of *MZezh1*^*ul3/ul3*^ embryos is observed. Scale bar is 500 μm. The numbers indicate the number of embryos with the displayed compared to the total number of embryos analyzed. (**B**) Expression analysis of *ezh1* in wild-type and *MZezh1*^*ul3/ul3*^ embryos and larvae at 1, 2, 3, 4 and 5 dpf quantified by RT-qPCR show an increase of *ezh2* expression in *MZezh1*^*ul3/ul3*^ mutants. Three independent experiments were performed and statistical analysis was conducted using Student’s t-test. *P < 0.05; **P < 0.01; ***P < 0.001.

Given that H3K27me2/3 levels are not decreased in *ezh1*^*ul3/ul3*^ mutant fish, we next tested if *ezh2* expression is changed in absence of *ezh1* function. At 48 hpf, *ezh2* expression is restricted to specific regions such as the pectoral fin buds, the optic tectum, the mid-hindbrain region, the branchial arches and the retina (Fig. 6A)^26,28^. Whole-mount *in situ* hybridization seems to show a slight increase in the *ezh2* signal in the pectoral fin buds and at both sides of the mid-hindbrain region in *MZezh1*^*ul3/ul3*^ embryos at 48 hpf when compared to wild-type (Fig. 6A). This increase in *ezh2* expression in *MZezh1*^*ul3/ul3*^ mutant was confirmed by RT-qPCR on mRNAs extracted from embryos from 1 to 5 dpf. Quantification of transcripts shows a significant increase in *ezh2* mRNA abundance in *MZezh1*^*ul3/ul3*^ mutants when compared to wild-type at 2-, 3-, 4- and 5 dpf (Fig. 6B).

### Ezh1 contributes to zebrafish development in absence of *ezh2*

We have previously generated an *ezh2*^+*/ul2*^ mutant line using the TALEN technology^26^. Ezh2-deficient zebrafish mutants present a normal body plan but die at around 12 dpf with defects in the intestine wall, due to enhanced cell death. In order to uncover the role of Ezh1 in zebrafish development, lines harboring mutations in both *ezh1* and *ezh2* genes were generated. Fish from the lines *ezh1;*^+*/ul3*^ *ezh2*^+*/ul2*^ and *ezh1;*^*ul3/ul3*^ *ezh2*^+*/ul2*^ are viable, fertile, healthy and do not show any phenotype.

To study the role of Ezh1 in absence of Ezh2, incross of *ezh1*;^*ul3/ul3*^ *ezh2*^+*/ul2*^ fish was performed and larvae were genotyped at 11 dpf. At this stage, we failed to identify larvae with the *ezh1*^*ul3/ul3*^; *ezh2*^*ul2/ul2*^ genotype out of 32 individuals checked, indicating that loss of maternal and zygotic *ezh1* expression could be responsible for a premature death of *ezh2*^*ul2/ul2*^ larvae.

**Figure 7:**
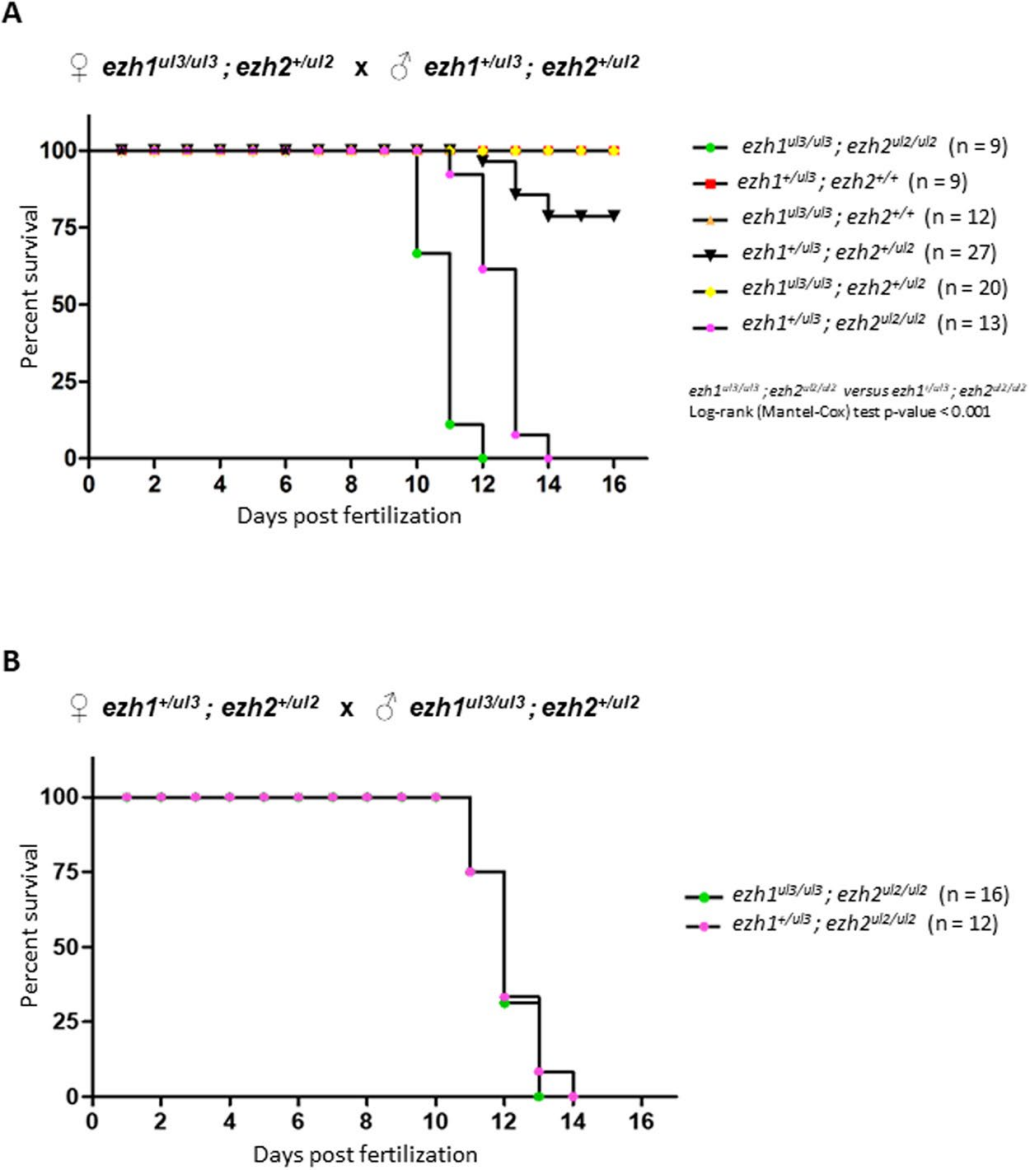
*Ezh1* contributes to zebrafish development in absence of ezh2. Kaplan-Meier survival curves over 16 days for siblings from the indicated genotypes from crosses between ♀*ezh1*^*ul3/ul3*^; *ezh2*^+*/ul2*^ and ♂*ezh1*^+*/ul3*^; *ezh2*^+*/ul2*^ (**A**) and from crosses between ♀*ezh1*^+*/ul3*^; *ezh2*^+*/ul2*^ and ♂*ezh1*^*ul3/ul3*^; *ezh2*^+*/ul2*^ (**B**). The number of fishes of each genotype is indicated. Statistical significance was assessed using a log-rank (Mantel-Cox) test.

To further investigate the effect of *ezh1* deficiency on the survival of *ezh2*^*ul2/ul2*^ larvae, we performed Kaplan-Meier survival curves. First, *ezh1*^*ul3/ul3*^; *ezh2*^+*/ul2*^ females were crossed with *ezh1*^+*/ul3*^; *ezh2*^+*/ul2*^ males. At 3dpf, siblings were genotyped and embryos were pooled into distinct tanks according to their genotype and their survival followed to generate Kaplan-Meier plots (Fig. 7A). As previously shown for *ezh2*^*ul2/ul2*^ mutants^26^, lethality of *ezh1;*^*+/ul3*^ *ezh2*^*ul2/ul2*^ fish occurs at around 12 dpf (50% survival: 13 dpf). In contrast, mutants lacking *ezh1* function (*MZezh1*^*ul3/ul3*^; *ezh2*^*ul2/ul2*^) are dying about two days earlier (50% survival: 11 dpf) indicating that maternal and/or zygotic *ezh1* expression contributes to zebrafish development in the absence of zygotic *ezh2* function. However, no morphological differences between *ezh1;*^+*/ul3*^ *ezh2*^*ul2/ul2*^ and *MZezh1*^*ul3/ul3*^; *ezh2*^*ul2/ul2*^ larvae could be observed.

To determine the role of maternal *ezh1* expression in the survival of *ezh2*-deficient larvae, a cross between *ezh1;*^+*/ul3*^ *ezh2*^+*/ul2*^ females and *ezh1*^*ul3/ul3*^; *ezh2*^+*/ul2*^ males was set up to generate paternal-zygotic (PZ) *PZezh1*^*ul3/ul3*^; *ezh2*^*ul2/ul2*^ mutants. In this situation, all embryos will benefit from *ezh1* maternal products. Figure 7B reveals that in presence of maternal *ezh1* products, *ezh1*^+*/ul3*^; *ezh2*^*ul2/ul2*^ and *ezh1*^*ul3/ul3*^; *ezh2*^*ul2/ul2*^ have similar survival rates indicating that *ezh1* products from maternal origin are crucial for larval development in absence of *ezh2*.

## Discussion

Considering that *Drosophila* and other invertebrates have a single gene coding for the H3K27me2/3 histone methyltransferase while mammalian genomes encode two orthologs, we searched for the phylogenetic origin of Ezh1 and Ezh2 in vertebrate evolution. We found that *ezh2*, but not *ezh1* is present in the sea lamprey genome suggesting that the vertebrate ancestors contained only one gene, *ezh2*, encoding this histone lysine methyltransferase activity. Ezh1 might have then arisen through *ezh2* gene duplication during evolution. This is in agreement with the fact that Ezh2 being the closest to its invertebrate homologs^11^.

In the cartilaginous fish, only *ezh2* was found in the elephant shark genome whereas both *ezh1* and *ezh2* were identified in the genomes of the whale shark and of the little skate. This implies that the current vertebrate phylogenic tree based on fossils and proposing that holocephalans and elasmobranchs diverged from a common ancestor distinct from the bony vertebrate ancestor should be revised. We propose that holocephalans including the elephant shark diverged before the appearance of a common ancestor of the elasmobranchs including the whale shark and the little skate and of the bony vertebrates including the ray-finned fish, the lobe-finned fish and the tetrapods in which the *ezh2* gene duplication occurred. Obviously, this hypothesis should be tested once genomic data from other lampreys, chimeras and sharks will be available.

A whole-genome duplication (Ts3R) and a subsequent massive loss of duplicated onhologs occurred in the teleost lineage^32,33^. As a consequence of these genomic events, some genes were differentially lost in different teleost species. For instance, Ring1 and Rnf2 are components of the PRC1 protein complex and are the E3 ubiquitin-protein ligases that mediate monoubiquitination of Lysine 119 of histone H2A (H2AK119ub1)^10,47^. Analysis of teleostean genomes revealed that both *ring1* and *rnf2* genes are present in the medaka genome, but *ring1* has been lost in zebrafish, in green spotted puffer (*Tetraodon nigroviridis*) and in Japanese puffer (*Takifugu rubripes*) suggesting that *ring1* and *rnf2* are redundant and that Rnf2 function alone is sufficient and could bypass Ring1 requirement^48^. A search for *ezh1* in 25 teleost species having their genome sequenced shows that the gene is present even in fish with particularly small genome size from the tetraodontidae family (e.g., the pufferfish *Takifugu rubripes* and *Tetraodon nigroviridis*). Since gene duplications can provide the raw material on which evolution can act, since they lead to redundant gene copies that are freed up to evolve novel gene functions^49^, one could then argue that Ezh1 and Ezh2 have at least slightly different functions. However, in the green spotted puffer (*Tetraodon nigroviridis*), a gene coding for *ezh1* could be identified (ENSTNIG00000011694) while *ezh2* seems to be absent. This shows that Ezh1 and Ezh2 are functionally redundant and it indicates that *ezh1* could bypass the *ezh2* loss in this pufferfish.

To question the function of *ezh1*, we use the zebrafish model and first investigate *ezh1* expression during development. Previous reports were not able to detect *ezh1* mRNAs in zygotes and embryos prior to maternal to zygotic transition neither by *in situ* hybridization^50,51^ nor by RNA sequencing in a large scale approach^52^, but we do. Using *in situ* hybridization, we could detect maternally loaded *ezh1* transcripts at the 1- to 2-cell stages, and the specificity of the *ezh1* probe was assessed by showing that *in situ* hybridization experiments on embryos from *ezh1*^*ul3/ul3*^ incrosses gave a reduced *ezh1* signal. Furthermore, RT-PCR assays followed by sequencing of the amplicon confirmed the presence of *ezh1* mRNAs in the zebrafish before the midblastula transition, even if the maternal *ezh1* mRNA levels seem to be low. Our identification of maternally deposited *ezh1* transcripts in zebrafish embryos then contradicts the results from previous studies^50–52^. A possible explanation to this discrepancy could be linked to the considerable genetic variation between zebrafish strains^53^. Indeed, in the course of our studies, we identified a polymorphism within our TU strain at the *ezh1* locus in the region of exon 2/intron 2-3 (Supplementary Fig. S7). Even if we have no evidence indicating that this polymorphism alters *ezh1* expression or mRNA stability, one might be tempted to speculate that interstrain genetic variability may affect gene expression especially for genes coding for non-essential proteins.

At 24 hpf, the *ezh1* mRNAs are ubiquitously distributed in the embryo but *ezh1* expression becomes more restricted in the developing brain and in the pectoral fin buds at 48 hpf. However, quantitative RT-qPCR reveals that *ezh1* mRNA levels increase during larval development between 24 hpf and 5 dpf, whereas *ezh2* mRNA levels decrease during the same period, as previously described^28^. These results parallel experiments performed in mouse suggesting that PRC2-Ezh2 complexes would exert their functions in proliferative tissues while PRC2-Ezh1 would have a more important role in more differentiated cells^13,17^.

To further investigate the Ezh1 function, we generated an *ezh1*-loss of function zebrafish line using the TALEN technology. Homozygous *ezh1*^*ul3/ul3*^ zebrafish are viable and fertile, indicating that maternal and zygotic *ezh1* products are dispensable to zebrafish development. It is similar to what was observed in mouse since *Ezh1*-deficient mice are viable, fertile and healthy^54–58^. Also, as in mammalian cells in culture where it has been shown that knockdown of *Ezh1* does not result in global reduction of H3K27me3 levels^13^, these global levels are not affected in zebrafish larvae deficient for *ezh1*. Investigation of *ezh2* expression in *ezh1*^*ul3/ul3*^ mutants reveals that *ezh2* levels are increased when *ezh1* expression is lost. This increase in *ezh2* expression in addition to the functional redundancy of Ezh1 and Ezh2, could account at least in part, for the absence of phenotype and for H3K27me2/3 levels unchanged in *MZezh1*^*ul3/ul3*^ mutant zebrafish.

Using Cre-mediated conditional mutagenesis in mouse, it has been shown that in absence of Ezh2 function, Ezh1 plays a role in hair follicle formation and maintenance^55^, in hepatic homeostasis and regeneration^57^, in skeletal growth^56^, as well as in the pathogenesis of hematopoietic malignancies^58^. Here, we show loss of maternal and zygotic *ezh1* expression impairs the survival of *ezh2*-deficent zebrafish larvae. Furthermore and consistent with the fact that *ezh1* transcripts are maternally provided, we show that *ezh1* maternal products are involved in this effect.

Although Ezh1 and Ezh2 have been shown to be functionally redundant *in vitro*^13,17^, it remains unclear whether this is the case in the context of tissue development and homeostasis. Here we show on one hand that *ezh1*^*ul3/ul3*^ are viable and fertile and on the other hand that loss of both maternal-zygotic *ezh1* and zygotic *ezh2* functions results in an earlier mortality of zebrafish larvae when compared to zygotic *ezh2*-deficient fish. These findings underscores the functional redundancy between Ezh2 and Ezh1 in controlling zebrafish larval development.

## Methods

### Database searches and analyses

Using the sequences of human and zebrafish Ezh1 and Ezh2 SET domains as search queries, TBLASTN analyses were performed using the NCBI (https://blast.ncbi.nlm.nih.gov/Blast.cgi), the Ensembl (http://www.ensembl.org/Multi/Tools/Blast?db = core), the EFish Genomics (https://efishgenomics.integrativebiology.msu.edu/blast_search/) or the Skatebase (http://skatebase.org/skateblast-skatebase%E2%80%8B/) Genome Servers. Amino acid sequences of protein domains of various species were obtained from SMART database (http://smart.embl-heidelberg.de/). Multiple sequence alignments were performed using Clustal Omega (https://www.ebi.ac.uk/Tools/msa/clustalo/) and 3D-structure analysis of EZH2 SET obtained from the Protein Data Bank (https://www.rcsb.org/) with Jalview (http://www.jalview.org/).

### Zebrafish maintenance, embryo preparation and animal ethics statements

Zebrafish (TU strain) were maintained at 27.5 °C in a 14/10 h light/dark cycle. The evening before spawning, males and females were separated into individual breeding tanks (Tecniplast). Spontaneous spawning occurred the following morning when the light turned on and the plastic divider removed. Embryos or larvae were then collected and staged according to Kimmel *et al*.^59^. The chorions were removed from embryos by the action of 1% pronase (Sigma) for 1 min. Zebrafish embryos or larvae were fixed overnight in 4% paraformaldehyde in PBS (phosphate-buffered saline, Invitrogen), dehydrated gradually to 100% methanol and kept at −20 °C.

Zebrafish were handled in compliance with local care regulations according to the French and European Union guidelines for the handling of laboratory animals (Directive 2010/63/EU of the European Parliament and of the Council of 22 September 2010 on the protection of animals used for scientific purposes). The experimental procedures carried out on zebrafish were reviewed and approved by the local Ethics Committee, CEEA 75 Nord Pas-de-Calais (APAFiS approval number 2018011722529804).

### TALEN design and assembly

A TALEN pair was designed to target exon 3 of *ezh1* using the online TAL Effector-Nucleotide Targeter tool (https://tale-nt.cac.cornell.edu/)^60^. The target site also contains a BamHI restriction site used to determine TALEN efficacy and for genotyping purposes based on restriction fragment length polymorphism analysis.

*Ezh1*-specific TALEN vectors were constructed using the TALEN Golden Gate assembly system described by Cermak *et al*.^46^. The TALEN expression backbones, pCS2TAL3DD and pCS2TAL3RR^20^, and the plasmids providing repeat variable diresidues (NI, HD, NG and NN recognizing A, C, T and G bases, respectively)^46^ for Golden Gate Cloning were obtained from Addgene.

### mRNA injection into zebrafish embryos

Capped mRNAs were synthetized using the SP6 mMESSAGE mMACHINE kit (Ambion) from linearized plasmid templates. mRNAs (50–100 pg) were injected into 1-cell zebrafish embryos using a FemtoJet microinjector (Eppendorf).

### Genotype analyses

To achieve genotyping, DNA was extracted using sodium hydroxide and Tris. Pieces of caudal fin, or paraformaldehyde-fixed embryos and larvae were incubated in 20 µL 50 mM NaOH and heated for 20 min at 95 °C. The tubes were then cooled to 4 °C and 2 µL of 1 M Tris-HCl, pH7.4 was added to neutralize the basic solution. Genotype analysis of *ezh1* was performed by PCR on 2.5 µL of samples using the primer set ezh1_5_4892fwd (5′-GGCTCTGTTCCAGTCAAATCGCCGT-3′) and ezh1_3_5202rev (5′-AGCTTTGGGAAATGGCGAGGCAAA-3′) followed by PCR product digestion with the BamHI restriction enzyme. Genotype analysis of *ezh2* was performed using the primer set TAL_ezh2_5′_S21Ac (5′-GGTATGGTTGTTGCAGTTCACAGAC-3′) and TAL_ezh2_3′_S21Ac (5′-AACACCAAACTCTACACAAGCAGCA-3′) and digestion with DdeI^26^.

Sequence determinations (GATC-Biotech, Germany) were performed after cloning of the PCR products into pCR2.1 -TOPO (Thermofisher) according to the manufacturer’s instructions.

### Whole-mount *in situ* hybridization

Antisense- and sense-RNA probes were synthesized with the DIG RNA Labeling Kit (SP6/T7) (Roche, 11175025910), according to manufacturer’s instructions. The cDNA clone MGC:152758 IMAGE:2639510 purchased at imaGenes GmbH (Berlin) was used as a template for *ezh2*. *ezh1* antisense and sense probes were generated using RT-PCR from total mRNA extracted from zebrafish larvae at 5 dpf using the RNeasy Mini Kit (Qiagen). After Reverse Transcription, cDNAs were amplified by PCR using the probe specific primers, coupled to the T7 sequence for forward primers and the SP6 sequence for reverse primers.

The primers used for *ezh1* probe generation were:

ISH_ezh1_F: TAATACGACTCACTATAGGGGAGGAAGCGACCACGAAACCACC

ISH_ezh1_R: GATTTAGGTGACACTATAGGGAGACCTGTTTGCTGTCCCAGT

*In situ* hybridization experiments were performed as previously described^61^. The embryos were imaged using a Leica MZ10F stereomicroscope equipped with a Leica DFC295 digital camera.

### Histone extraction and western blot analysis

Histone extraction and Western blot analyses were performed from 10 larvae as described previously^62^.

Primary antibodies used were mouse anti-H3K27me3 (1:1,000; ab6002, Abcam), rabbit anti-H3K27me2 (1:500; ab24684, Abcam) and rabbit anti-H3 (1:5,000; ab1791, Abcam). The secondary antibodies were peroxidase conjugated anti-mouse antibody (1:10,000; 115-035-003, Jackson ImmunoResearch) and peroxidase conjugated anti-rabbit antibody (1:10,000; 711-035-152, Jackson ImmunoResearch).

### RNA extraction and RT-PCR

Total RNAs were purified from 1 hpf, 6, hpf, 1 dpf, 2 dpf, 3 dpf, 4 dpf and 5 dpf wild-type and mutant embryos or larvae using Trizol as previously described^62^. cDNA was synthesized using Superscript III (18080-044, Invitrogen) according to manufacturer’s instructions. About 40 embryos were used in total RNA extractions and 1 µg total RNAs were used to perform the reverse transcription experiments. Primers used were:

Dr_ezh1_cDNA_5a:5′-CGTCTAGTGAGGTCTGAGGATG-3′

Dr_ezh1_cDNA_3a: 5′-CCTCGTCCTGTTCCAACACTTC-3′

ZA027_ezh2_cDNA_5:5′-GAGGTGAAAGGACCCTCTACC-3′

ZA027_ezh2_cDNA_3:5′-CTCAGTTTCCATTCCTGATTTAAG-3′

Dr_cDNA_ube2a_F: 5′-TGACTGTTGACCCACCTTACAG-3′

Dr_cDNA_ube2a_R: 5′-CAAATAAAAGCAAGTAACCCCG-3′

Sequence determination (GATC-Biotech, Germany) was performed after cloning of the PCR products into pCR2.1-TOPO (Thermofisher) following the manufacturer’s instructions.

PCR reactions were performed as follow: 95 °C 4 min, [95 °C 45 sec, 55 °C 45 sec, 72 °C 1 min] 40 cycles, 72 °C 10 min.

The quantitative qPCR reaction was performed in triplicate using a Bio-Rad CFX96 Real-Time System using SYBR Green Supermix (Bio-Rad). Relative mRNA expression of each gene was normalized to *ube2a* levels.

### Kaplan-Meier analysis

At 3 dpf, the tip of the caudal fin was transected within the pigment gap distal to the circulating blood for genotyping purposes^62^. The embryos were pooled into separate 1-liter tanks according to their genotype and placed into an incubator set up at 28 °C. Larvae were fed from 5 dpf on with early stage zebrafish nutrition (Gemma Micro ZF 75, Planktovie) three times per day and regularly checked directly in the tanks using a Leica EZ4 stereomicroscope with no or minimal manipulation, during a period of 16 days post fertilization. Larvae were declared dead when heart beats could not be detected under stereomicroscopic examination. Kaplan-Meier plots were generated and log-rank (Mantel-Cox) test p-values calculated with Prism (GraphPad Software).

## Supplementary information

Supplementary Dataset 1

## Data Availability

All relevant data are within the paper and its Supplementary Information Files.
