# Supplementary material for: Ezh1 arises from Ezh2 gene duplication but its function is not required for zebrafish development
Source: Sci Rep. 2019 Mar 13;9:4319. doi: 10.1038/s41598-019-40738-9 (PMC6416316; doi:10.1038/s41598-019-40738-9)
Supplement: Supplementary file 1 — Supplementary Dataset 1 [file 41598_2019_40738_MOESM1_ESM.pdf]

Ezh1 arises from Ezh2 gene duplication but its function is not required for zebrafish development

Pamela Völkel, Aurélie Bary, Ludivine Raby, Anaïs Chapart, Barbara Dupret, Xuefen Le Bourhis and Pierre-Olivier Angrand *

ADDITIONAL INFORMATION

Supplementary Table S1. List of the accession numbers corresponding to ezh1 genes in fish. The Databases used for TBLASTN searches are indicated. Teleosts are listed in the dashed box. * *Ezh2* was not found in the genome of the green spotted puffer *T. nigroviridis*.

Supplementary Figure S1. Multiple protein alignment of pre-SET and SET domains of Ezh2 and Ezh1 from human (Hs: *Homo sapien*), mouse (Mm: *Mus musculus*), mallard (Ap: *Anas platyrhynchos*) turkey (Mg: *Meleagris gallopavo*), green anole (Ac: *Anolis corolinensis*), bearded dragon (Pv: *Pogona vitticeps*), western clawed frog (Xt: *Xenopus tropicalis*), high Himalaya frog (Np: *Nanorana parkeri*), zebrafish (Dr: *Danio rerio*) and medaka (Ol: *Oryzia latipes*). Secondary structure assignments are depicted above the alignment. Residues which coordinate zinc are underlined and in blue. Amino-acids distinguishing Ezh1 from Ezh2 are in red and lighted up with an arrow. The K661R and V662I substitutions specific to Ezh1 from ray-finned fishes are in blue and underlined.

Supplementary Figure S2. Classical phylogeny of vertebrates. Holocephalans and elasmobranchs have diverged from a common ancestor. The major shared features of vertebrate taxa are indicated.

Supplementary Figure S3. Ezh1 in elasmobranchs. (**A**) Multiple protein alignment of Ezh2 and Ezh1 SET domains from human (Hs: *Homo sapien*), mouse (Mm: *Mus musculus*), zebrafish (Dr: *Danio rerio*) and the predicted Ezh1 SET domain encoded by LOC109922287 from the whale shark (Rt: *Rhincodon typus*). The Ezh1 SET domain from the whale shark contains the Ezh1-specific substitutions I631T, C668S and M705V (in green), but not I650L (in red, underlined). (**B**) Multiple protein alignment of Ezh2 and Ezh1 SET domains from human (Hs: *Homo sapien*), mouse (Mm: *Mus musculus*), zebrafish (Dr: *Danio rerio*) and the predicted Ezh1 SET domain encoded by the partial transcript LS-transcriptB2-ctg80080 (ctg80080) from the little skate (Le: *Leucoraja eriacea*). The Ezh1 SET domain from the whale shark contains the Ezh1-specific substitutions C668S and M705V (in green), but not I650L (in red, underlined).

Supplementary Figure S4. Maternally loaded ezh1 transcripts revealed by in situ hybridization. (**A**) Schematic representation of the zebrafish *ezh1* locus with coding and untranslated sequences depicted as solid and open boxes, respectively. The location of primers used to generate the sense and antisense probes in exons 10 and 15 is shown in red (**B**) Parallel *in situ* hybridizations at the 1- to 2-cell stage reveal a clear signal with the antisense RNA probe on wild-type embryos, a weak signal with the antisense RNA probe on *MZezh1*$^{ul3/ul3}$ embryos from an *ezh1*$^{ul3/ul3}$ incross and no signal with the sense RNA probe on wild-type embryos.

Supplementary Figure S5. Maternally loaded ezh1 transcripts identified by RT-PCR and sequencing of the amplicon. (**A**) Schematic representation of the transcripts produced at the *ezh1* locus. *ezh1-001* (ENSDART00000039170.8) corresponds to the canonical *ezh1* mRNA,

while *ezh1-002* (ENSDART00000101965.6) is a shorter predicted transcript. The location of the primers used in RT-PCR experiments is shown as red arrowheads. Amplification of the *ezh1* (*ezh1-001*) mRNA by RT-PCR is expected to give a 470 bp amplicon. Due to the presence of intron 4-5 in the *ezh1-002* transcript, its amplification by RT-PCR gives an 871 bp DNA product. (**B**) RT-PCR analysis showing the detection of a 470 bp product from RNAs extracted from zebrafish embryos at 1 hpf. The reverse transcriptase (RT) was included (+) or not (-) in the reaction as indicated. After cloning and sequencing, a BLAST analysis reveals that the amplicon corresponds to the *ezh1* cDNA sequence (**C**) having the intron 4 spliced out (**D**).

Supplementary Figure S6. Predicted product of the ezh1 mutant allele. (**A**) Schematic representation of the zebrafish Ezh1 protein. Red, violet and brown motifs correspond to SANT (SMART: SM00717), CXC (SMART: SM01114) and SET (SMART: SM00317) domains, respectively. Size of the protein is indicated. (**B**) Amino-acid sequence of the wild-type zebrafish Ezh1 protein. Peptides coding for the conserved SANT, CXC and SET domains are indicated in red, violet and brown, respectively. (**C**) Predicted amino-acid sequence of the protein encoded by the *ezh1*^{ul3} mutant allele. The amino-acid in common with the wild-type allele are indicated in blue. The point where the frameshift occurs is shown with the arrow.

Supplementary Figure S7. Identification of an ezh1 polymorphism in the TU zebrafish strain. (**A**) Schematic representation of the zebrafish *ezh1* locus with coding and untranslated sequences depicted as solid and open boxes, respectively. The location of primers used for genomic DNA amplification by PCR is shown in red. (**B**) Sequence of the primers used. TAL_ezh1_5c and TAL_ezh1_3b amplify a region C in exon 2 / intron 2-3, ezh1_5_4892fwd and ezh1_3_5202rev amplify a region A in exon 3 / intron 3-4, and ezh1_5_27681fwd and ezh1_3_28067rev amplify a region B in intron 14-15 / exon 15. (**C**) PCR amplification from genomic DNA extracted from 2 independent fish (#1 and #2) from the TU strain. Fragment C could not be amplified from the fish #2, whereas other fragment could be, indicating that primers TAL_ezh1_5c or/and TAL_ezh1_3b do not recognize complementary sequences in the fish #2 genome. In October to December 2013, Fragment C could not be amplified by PCR from 11 fish out of 22 fish analyzed from our TU strain stock.

Supplementary Figure S8. Generation of ezh1 mutant zebrafish using the TALEN technology. Genomic DNA from eleven ezh1 TALEN injected embryos (E1-11) and an unjected (Control) embryo were extracted at 3dpf. The TALEN targeted DNA region is amplified by PCR and subjected to BamHI digestion. The TAL-ezh1 injected embryo contains undigested material (arrow at 311 bp), indicating that the BamHI diagnostic restriction site has been disrupted. MW: molecular weight. This gel was used to generate Fig. 4B.

	Name	Family	Ezh1 Gene ID	Database
Dr	Zebrafish (*Danio rerio*)	Cyprinidae	ID: 664754	NCBI
Cc	Common carp (*Cyprinus carpio*)	Cyprinidae	LOC109102790<br>LOC109057529	NCBI
Ol	Medaka (*Oryzia latipes*)	Adrianichthyidae	ID: 101166385	NCBI
Mz	Zebra mbuna (*Maylandia zebra*)	Cichlidae	ID: 10147430	NCBI
On	Tilapia (*Oreochromis niloticus*)	Cichlidae	ID: 100695015	NCBI
Tn	Green spotted puffer (*Tetraodon nigroviridis*)*	Tetraodontidae	ENSTNIG00000011694	Ensembl
Tr	Japanese puffer (*Takifugu rubripes*)	Tetraodontidae	ID: 101070195	NCBI
Xm	Platyfish (*Xiphophorus maculatus*)	Poeciliidae	ID: 102228014	NCBI
Pf	Amazon molly (*Poecilia formosa*)	Poeciliidae	LOC103142220	NCBI
Pr	Guppy (*Poecilia reticulata*)	Poeciliidae	ID: 103468644	NCBI
Nf	Turquoise killifish (*Nothobranchius furzeri*)	Nothobranchiidae	ID: 107378461	NCBI
Fh	Mummichog (*Fundulus heteroclitus*)	Fundulidae	ID: 105937399	NCBI
Am	Cave fish (*Astyanax mexicanus*)	Characidae	ID: 103044086	NCBI
Gm	Cod (*Gadus morhua*)	Gadidae	ENSGMOG00000015628	Ensembl
Ga	Stickleback (*Gasterosteus aculeatus*)	Gasterosteidae	ENSGACG00000008492	Ensembl
Km	Mangrove rivulus (*Kryptolebias marmoratus*)	Rivulinae	ID: 108243802	NCBI
Cs	Tongue sole (*Cynoglossus semilaevis*)	Cynoglossidae	ID: 103393149	NCBI
El	Northern pike (*Esox lucius*)	Esocidae	ID: 105013194	NCBI
Ip	Channel catfish (*Ictalurus punctatus*)	Ictaluridae	ID: 108279279	NCBI
Ee	Electric eel (*Electrophorus electricus*)	Gymnotidae	C7984347.g1.t1	EFISH
Lcr	Large yellow croaker (*Larimichthys crocea*)	Sciaenidae	ID: 104923910	NCBI
Sf	Asian arowana (*Scleropages formosus*)	Osteoglossidae	LOC108928200<br>LOC108941744	NCBI
Ss	Atlantic salmon (*Salmo salar*)	Salmonidae	LOC106607313<br>LOC106601045	NCBI
Om	Rainbow trout (*Oncorhynchus mykiss*)	Salmonidae	LOC110486277<br>LOC110538212	NCBI
Ok	Coho salmon (*Oncorhynchus kisutch*)	Salmonidae	LOC109898438<br>LOC109891924	NCBI
Lo	Spotted gar (*Lepisosteus oculatus*)	Lepisosteidae	ID: 102693111	NCBI
Lc	Coelacanth (*Latimeria chalumnae*)	Latimeriidae	ID: 102364190	NCBI
Rt	Whale shark (*Rhincodon typus*)	Rhincodontidae	LOC109924829 (N-term)<br>LOC109922287 (C-term)	NCBI
Le	Little skate (*Leucoraja erinacea*)	Rajidae	ctg80080	SkateBase
Cm	Elephant shark (*Callorhinchus milii*)	Callorhinchidae	**Not found**	NCBI
Pm	Sea lamprey (*Petromyzon marinus*)	Petromyzontidae	**Not found**	Ensembl

Völkel et al. – Supplementary Table S1

```
                            α1                                   α2
Hs_EZH2    QPCDHPRQPCDSSCPCVIAQNFCEKFCQCSSECQNRFPGCRCKAQCNTKQCPCYLAVRECDPDLCLTCGAADHWDSKNVS   605
Mm_Ezh2    QPCDHPRQPCDSSCPCVIAQNFCEKFCQCSSECQNRFPGCRCKAQCNTKQCPCYLAVRECDPDLCLTCGAADHWDSKNVS   600
Mg_Ezh2    QPCDHPRQPCDSSCPCVIAQNFCEKFCQCSSECQNRFPGCRCKAQCNTKQCPCYLAVRECDPDLCLTCGAADHWDSKNVS   600
Ap_Ezh2    QPCDHPRQPCDSSCPCVIAQNFCEKFCQCSSECQNRFPGCRCKAQCNTKQCPCYLAVRECDPDLCLTCGAADHWDSKNVS   592
Ac_Ezh2    QPCDHPRQPCDNSCPCVIAQNFCEKFCQCSSECQNRFPGCRCKAQCNTKQCPCYLAVRECDPDLCLTCGAADHWDSKNVS   636
Pv_Ezh2    QPCDHPRQPCDSSCPCVIAQNFCEKFCQCSSECQNRFPGCRCKAQCNTKQCPCYLAVRECDPDLCLTCGAADHWDSKNVS   639
Xt_Ezh2    QPCDHPRQPCDSSCPCVIAQNFCEKFCQCSSECQNRFPGCRCKAQCNTKQCPCYLAVRECDPDLCLTCGAADHWDSKNVS   602
Np_Ezh2    QPCDHPRQPCDGSCPCVIAQNFCEKFCQCSSECQNRFPGCRCKAQCNTKQCPCYLAVRECDPDLCLTCGAADHWDSKNVS   603
Dr_Ezh2    QPCDHPRQPCDSSCPCVTAQNFCEKFCQCSSECQNRFPGCRCKAQCNTKQCPCYLAVRECDPDLCLTCGAAEHWDSKNVS   614
Ol_Ezh2    QPCDHPRQPCDSSCPCVTAQNFCEKFCQCSSECQNRFPGCRCKAQCNTKQCPCYLAVRECDPDLCLTCGAADHWDSKNVS   614

Hs_EZH1    QPCDHPDRPCDSTCPCIMTQNFCEKFCQCNPDCQNRFPGCRCKTQCNTKQCPCYLAVRECDPDLCLTCGASEHWDCKVVS   601
Mm_Ezh1    QPCDHPDRPCDSTCPCIMTQNFCEKFCQCSPDCQNRFPGCRCKTQCNTKQCPCYLAVRECDPDLCLTCGASEHWDCKVVS   604
Mg_Ezh1    QPCDHPEHPCDSSCPCIMTQNFCEKFCQCNPDCQNRFPGCRCKTQCNTKQCPCYLAVRECDPDLCLTCGASEHWDCKVVS   600
Ap_Ezh1    QPCDHPEHPCDSSCPCIMTQNFCEKFCQCNPDCKNRFPGCRCKTQCNTKQCPCYLAVRECDPDLCLTCGASEHWDCKVVS   520
Ac_Ezh1    QPCDHPDHPCDNSCPCIMTQNFCEKFCQCNPDCQNRFPGCRCKTQCNTKQCPCYLAVRECDPDLCLTCGAAEHWDSKVVP   608
Pv_Ezh1    QPCDHPEHPCDSSCPCIMTQNFCEKFCQCNPDCQNRFPGCRCKTQCNTKQCPCYLAVRECDPDLCLTCGASEHWDSKVVS   620
Xt_Ezh1    QPCDHPDHPCDSTCPCIMTQNFCEKFCQCNPDCQNRFPGCRCKTQCNTKQCPCYLAVRECDPDLCLTCGASEHWDSKVVS   602
Np_Ezh1    QPCDHPDHPCDSTCPCIMTQNFCEKFCQCNPDCQNRFPGCRCKTQCNTKQCPCYLAVRECDPDLCLTCGASEHWDSKIVS   564
Dr_Ezh1    QPCDHPEHPCDSSCPCVITQNFCEKFCQCDRECQNRFPGCRCKTQCNTKQCPCYLAVRECDPDLCMTCGAADHWDSKQVS   610
Ol_Ezh1    QPCDHPDHPCDNSCPCVMTQNFCEKFCQCENECQNRFPGCRCKTQCNTKQCPCYLAVRECDPDLCMTCGAADHWDSKVVT   620
           ****** :***.:***: :**********. :*:*********:*************** ****:****::***.* *

              α3      β1        β2         β3      β4     α4               β5      β6
Hs_EZH2    CKNCSIQRGSKKHLLLAPSDVAGWGIFIKDPVQKNEFISEYCGEIISQDEADRRGKVYDKYMCSFLFNLNNDFVVDATRK   685
Mm_Ezh2    CKNCSIQRGSKKHLLLAPSDVAGWGIFIKDPVQKNEFISEYCGEIISQDEADRRGKVYDKYMCSFLFNLNNDFVVDATRK   680
Mg_Ezh2    CKNCSIQRGSKKHLLLAPSDVAGWGIFIKDPVQKNEFISEYCGEIISQDEADRRGKVYDKYMCSFLFNLNNDFVVDATRK   680
Ap_Ezh2    CKNCSIQRGSKKHLLLAPSDVAGWGIFIKDPVQKNEFISEYCGEIISQDEADRRGKVYDKYMCSFLFNLNNDFVVDATRK   672
Ac_Ezh2    CKNCSIQRGSKKHLLLAPSDVAGWGIFIKDPVQKNEFISEYCGEIISQDEADRRGKVYDKYMCSFLFNLNNDFVVDATRK   716
Pv_Ezh2    CKNCSIQRGSKKHLLLAPSDVAGWGIFIKDPVQKNEFISEYCGEIISQDEADRRGKVYDKYMCSFLFNLNNDFVVDATRK   719
Xt_Ezh2    CKNCSIQRGSKKHLLLAPSDVAGWGIFIKDPVQKNEFISEYCGEIISQDEADRRGKVYDKYMCSFLFNLNNDFVVDATRK   682
Np_Ezh2    CKNCSIQRGSKKHLLLAPSDVAGWGIFIKEPVQKNEFISEYCGEIISQDEADRRGKVYDKYMCSFLFNLNNDFVVDATRK   683
Dr_Ezh2    CKNCSIQRGAKKHLLLAPSDVAGWGIFIKEPVQKNEFISEYCGEIISQDEADRRGKVYDKYMCSFLFNLNNDFVVDATRK   694
Ol_Ezh2    CKNCSIQRGAKKHLLLAPSDVAGWGIFIKEPVQKNEFISEYCGEIISQDEADRRGKVYDKYMCSFLFNLNNDFVVDATRK   694

Hs_EZH1    CKNCSIQRGLKKHLLLAPSDVAGWGTFIKESVQKNEFISEYCGELISQDEADRRGKVYDKYMSSFLFNLNNDFVVDATRK   681
Mm_Ezh1    CKNCSIQRGLKKHLLLAPSDVAGWGTFIKESVQKNEFISEYCGELISQDEADRRGKVYDKYMSSFLFNLNNDFVVDATRK   684
Mg_Ezh1    CKNCSIQRGLKKHLLLAPSDVAGWGTFIKESVQKNEFISEYCGELISQDEADRRGKVYDKYMSSFLFNLNNDFVVDATRK   680
Ap_Ezh1    CKNCSIQRGLKKHLLLAPSDVAGWGTFIKESVQKNEFISEYCGELISQDEADRRGKVYDKYMSSFLFNLNNDFVVDATRK   700
Ac_Ezh1    CKNCSIQRGLKKHLLLAPSDVAGWGTFIKEAVQKNEFISEYCGELISQDEADRRGKVYDKYMSSFLFNLNNDFVVDATRK   688
Pv_Ezh1    CKNCSIQRGLKKHLLLAPSDVAGWGTFIKEAVQKNEFISEYCGELISQDEADRRGKVYDKYMSSFLFNLNNDFVVDATRK   700
Xt_Ezh1    CKNCSIQRGLKKHLLLAPSDVAGWGTFIKESVQKNEFISEYCGELISQDEADRRGKVYDKYMSSFLFNLNNDFVVDATRK   682
Np_Ezh1    CKNCSIQRGLKKHLLSAPSDVAGWGTFIKESVQKNEFISEYCGELISQDEADRRGKVYDKYMSSFLFNLNNDYVVDATRK   644
Dr_Ezh1    CKNCSIQRGLKKHLLLAPSDVAGWGTFIKEPVQKNEFISEYCGELISQDEADRRGRIYDKYMSSFLFNLNNDFVVDATRK   690
Ol_Ezh1    CKNCSIQRGLKKHLLLAPSDVAGWGTFIKEPVQKNEFISEYCGELISQDEADRRGRIYDKYMSSFLFNLNNDFVVDATRK   700
           ********* ***** ********* ***: *************:**********::*****.*********:*******

              α5          β7         β8
Hs_EZH2    GNKIRFANHSVNPNCYAKVMMVNGDHRIGIFAKRAIQTGEELFFDYRYSQADALKYVGIEREMEIP   751
Mm_Ezh2    GNKIRFANHSVNPNCYAKVMMVNGDHRIGIFAKRAIQTGEELFFDYRYSQADALKYVGIEREMEIP   746
Mg_Ezh2    GNKIRFANHSVNPNCYAKVMMVNGDHRIGIFAKRAIQTGEELFFDYRYSQADALKYVGIEREMEIP   746
Ap_Ezh2    GNKIRFANHSVNPNCYAKVMMVNGDHRIGIFAKRAIQTGEELFFDYRYSQADALKYVGIEREMEIP   738
Ac_Ezh2    GNKIRFANHSVNPNCYAKVMMVNGDHRIGIFAKRAIQTGEELFFDYRYSQADALKYVGIEREMEIP   782
Pv_Ezh2    GNKIRFANHSVNPNCYAKVMMVNGDHRIGIFAKRAIQTGEELFFDYRYSQADALKYVGIEREMEIP   784
Xt_Ezh2    GNKIRFANHSVNPNCYAKVMMVNGDHRIGIFAKRAIQTGEELFFDYRYSQADALKYVGIEREMEIP   748
Np_Ezh2    GNKIRFANHSVNPNCYAKVMMVNGDHRIGIFAKRAIQTGEELFFDYRYSQADALKYVGIEREMEIP   749
Dr_Ezh2    GNKIRFANHSVNPNCYAKVMMVNGDHRIGIFAKRAIQTGEELFFDYRYSQADALKYVGIEREMEIP   760
Ol_Ezh2    GNKIRFANHSVNPNCYAKVMMVSGDHRIGIFAKRAIQTGEELFFDYRYSQADALKYVGIEREMEIA   760

Hs_EZH1    GNKIRFANHSVNPNCYAKVVMVNGDHRIGIFAKRAIQAGEELFFDYRYSQADALKYVGIERETDVL   747
Mm_Ezh1    GNKIRFANHSVNPNCYAKVVMVNGDHRIGIFAKRAIQAGEELFFDYRYSQADALKYVGIERETDVF   750
Mg_Ezh1    GNKIRFANHSVNPNCYAKVVMVNGDHRIGIFAKRAIQAGEELFFDYRYSQADALKYVGIERETDII   746
Ap_Ezh1    GNKIRFANHSVNPNCYAKVVMVNGDHRIGIFAKRAIQAGEELFFDYRYSQADALKYVGIERETDII   666
Ac_Ezh1    GNKIRFANHSVNPNCYAKVVMVNGDHRIGIFAKRAIQAGEELFFDYRYSQADALKYVGIEREGDIV   753
Pv_Ezh1    GNKIRFANHSVNPNCYAKVVMVNGDHRIGIFAKRAIQAGEELFFDYRYSQADALKYVGIERETDII   766
Xt_Ezh1    GNKIRFANHSVHPNCYAKVVMVNGDHRIGIFAKRTIQAGEELFFDYRYSQADALKYVGIERETDIM   748
Np_Ezh1    GNKIRFANHSLNPNCYAKVVMVNGDHRIGIFAKRAIQAGEELFFDYRYSQADALKYVGIERESDAV   710
Dr_Ezh1    GNKIRFANHSVNPNCYAKVVMVNGDHRIGIFAKRAIQQGEELFFDYRYSQADALKYVGIEREIEIV   756
Ol_Ezh1    GNKIRFANHSVNPNCYAKVVMVNGDHRIGIFAKRAILQGEELFFDYRYSQADALKYVGIEREVDMT   766
           **********::*******:**.***********:*  ************ *********** :
```

Völkel et al. – Supplementary Figure S1

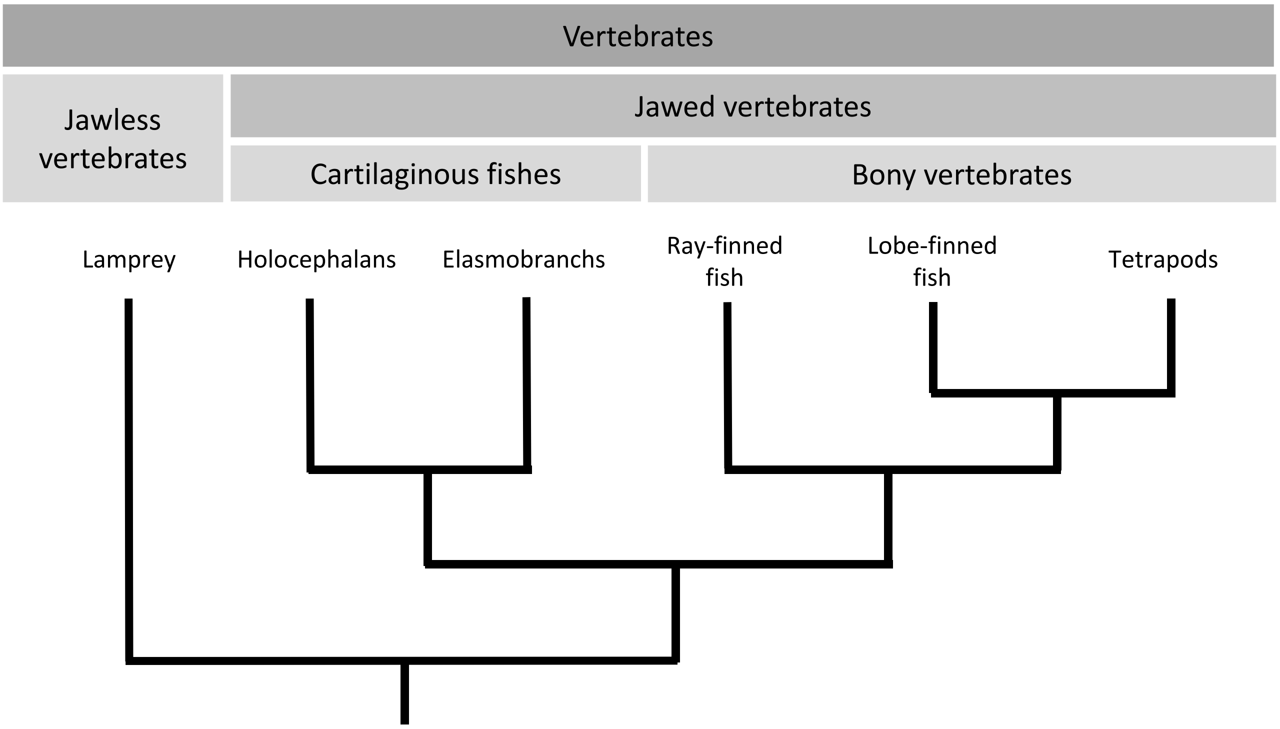

Völkel et al. – Supplementary Figure S2

A Ezh1-like sequence in the whale shark (*Rhincodon typus*)

```
Hs_EZH2_SET         KHLLLAPSDVAGWGIFIKDPVQKNEFISEYCGEIISQDEADRRGKVYDKYMCSFLFNLNN      676
Mm_Ezh2_SET         KHLLLAPSDVAGWGIFIKDPVQKNEFISEYCGEIISQDEADRRGKVYDKYMCSFLFNLNN      671
Dr_Ezh2_SET         KHLLLAPSDVAGWGIFIKEPVQKNEFISEYCGEIISQDEADRRGKVYDKYMCSFLFNLNN      685
Rt_LOC109922287     QHLLIAPSDVAGWGTFIKESVQKNEFISEYCGEIISQDEADRRGKVYDKYMSSFLFNLNN      -
Hs_EZH1_SET         KHLLLAPSDVAGWGTFIKESVQKNEFISEYCGELISQDEADRRGKVYDKYMSSFLFNLNN      672
Mm_Ezh1_SET         KHLLLAPSDVAGWGTFIKESVQKNEFISEYCGELISQDEADRRGKVYDKYMSSFLFNLNN      675
Dr_Ezh1_SET         KHLLLAPSDVAGWGTFIKEPVQKNEFISEYCGELISQDEADRRGRIYDKYMSSFLFNLNN      681
                    ************** ***: *************:**********::*****.********

Hs_EZH2_SET         DFVVDATRKGNKIRFANHSVNPNCYAKVMMVNGDHRIGIFAKRAIQTGEELFFDYRYSQA      723
Mm_Ezh2_SET         DFVVDATRKGNKIRFANHSVNPNCYAKVMMVNGDHRIGIFAKRAIQTGEELFFDYRYSQA      718
Dr_Ezh2_SET         DFVVDATRKGNKIRFANHSVNPNCYAKVMMVNGDHRIGIFAKRAIQTGEELFFDYRYSQA      732
Rt_LOC109922287     DFVVDATRKGNKIRFANHSVNPNCYAKVVMVNGDHRIGIFAKRMIHTGEELFFDYRYSQA      -
Hs_EZH1_SET         DFVVDATRKGNKIRFANHSVNPNCYAKVVMVNGDHRIGIFAKRAIQAGEELFFDYRYSQA      719
Mm_Ezh1_SET         DFVVDATRKGNKIRFANHSVNPNCYAKVVMVNGDHRIGIFAKRAIQAGEELFFDYRYSQA      722
Dr_Ezh1_SET         DFVVDATRKGNKIRFANHSVNPNCYAKVVMVNGDHRIGIFAKRAIQQGEELFFDYRYSQA      728
                    ****************************:************** ** *************

Hs_EZH2_SET         DA        725
Mm_Ezh2_SET         DA        720
Dr_Ezh2_SET         DA        734
Rt_LOC109922287     DA        -
Hs_EZH1_SET         DA        721
Mm_Ezh1_SET         DA        724
Dr_Ezh1_SET         DA        730
                    **
```

B Ezh1-like sequence in the little skate (*Leucoraja erinacea*)

```
Hs_EZH2_SET         KHLLLAPSDVAGWGIFIKDPVQKNEFISEYCGEIISQDEADRRGKVYDKYMCSFLFNLNN      676
Mm_Ezh2_SET         KHLLLAPSDVAGWGIFIKDPVQKNEFISEYCGEIISQDEADRRGKVYDKYMCSFLFNLNN      671
Dr_Ezh2_SET         KHLLLAPSDVAGWGIFIKEPVQKNEFISEYCGEIISQDEADRRGKVYDKYMCSFLFNLNN      685
Le_ctg80080         ---------------------------------IISQDEADRRGKVYDKYMSSFLFNLNN      -
Hs_EZH1_SET         KHLLLAPSDVAGWGTFIKESVQKNEFISEYCGELISQDEADRRGKVYDKYMSSFLFNLNN      672
Mm_Ezh1_SET         KHLLLAPSDVAGWGTFIKESVQKNEFISEYCGELISQDEADRRGKVYDKYMSSFLFNLNN      675
Dr_Ezh1_SET         KHLLLAPSDVAGWGTFIKEPVQKNEFISEYCGELISQDEADRRGRIYDKYMSSFLFNLNN      681
                                                    ::**********::*****.********

Hs_EZH2_SET         DFVVDATRKGNKIRFANHSVNPNCYAKVMMVNGDHRIGIFAKRAIQTGEELFFDYRYSQA      723
Mm_Ezh2_SET         DFVVDATRKGNKIRFANHSVNPNCYAKVMMVNGDHRIGIFAKRAIQTGEELFFDYRYSQA      718
Dr_Ezh2_SET         DFVVDATRKGNKIRFANHSVNPNCYAKVMMVNGDHRIGIFAKRAIQTGEELFFDYRYSQA      732
Le_ctg80080         DFVVDATRKGNKIRFANHSVNPNCYAKVVMVNGDHRIGIFAKRGIQAGDELFFDYRYSLA      -
Hs_EZH1_SET         DFVVDATRKGNKIRFANHSVNPNCYAKVVMVNGDHRIGIFAKRAIQAGEELFFDYRYSQA      719
Mm_Ezh1_SET         DFVVDATRKGNKIRFANHSVNPNCYAKVVMVNGDHRIGIFAKRAIQAGEELFFDYRYSQA      722
Dr_Ezh1_SET         DFVVDATRKGNKIRFANHSVNPNCYAKVVMVNGDHRIGIFAKRAIQQGEELFFDYRYSQA      728
                    ****************************:************** ** *************

Hs_EZH2_SET         DA        725
Mm_Ezh2_SET         DA        720
Dr_Ezh2_SET         DA        734
Le_ctg80080         DA        -
Hs_EZH1_SET         DA        721
Mm_Ezh1_SET         DA        724
Dr_Ezh1_SET         DA        730
                    **
```

Völkel et al. – Supplementary Figure S3

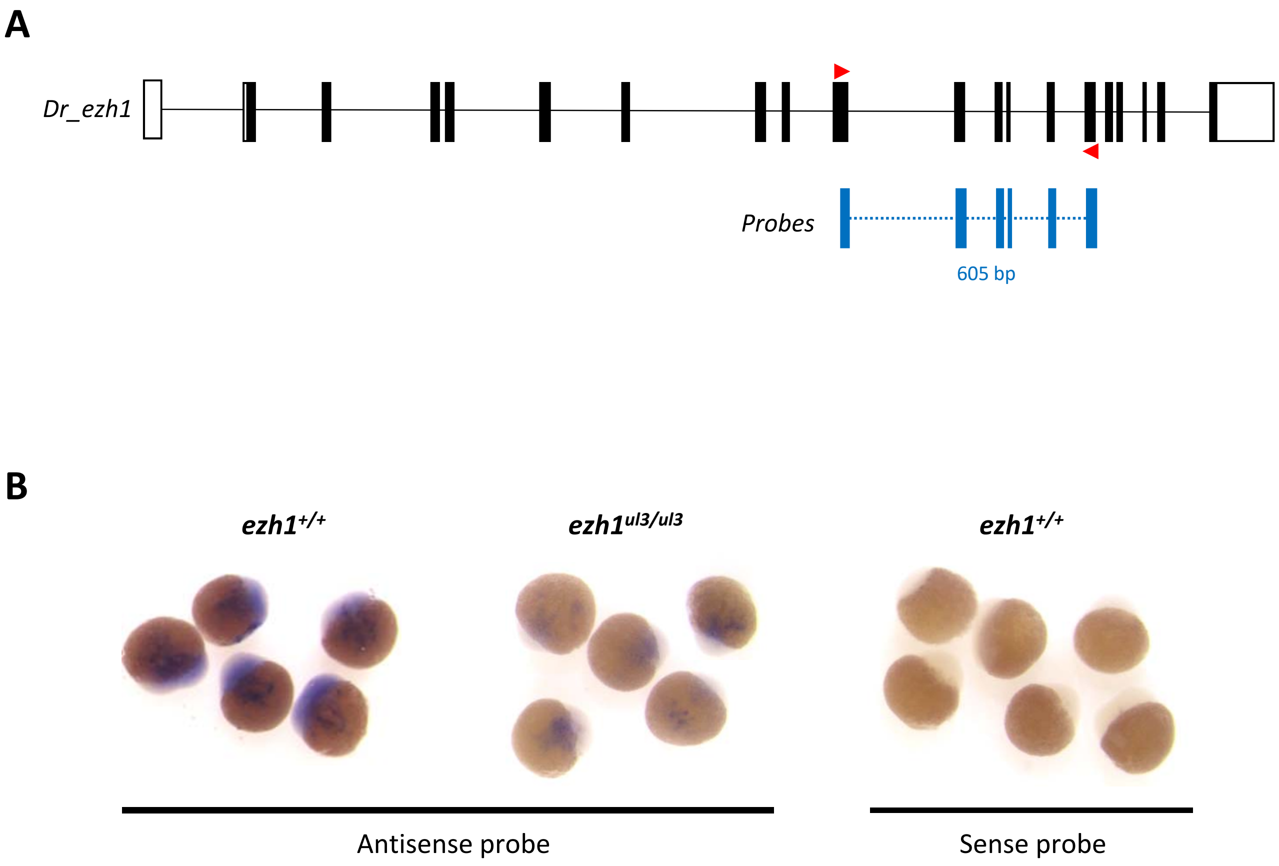

Völkel et al. – Supplementary Figure S4

A

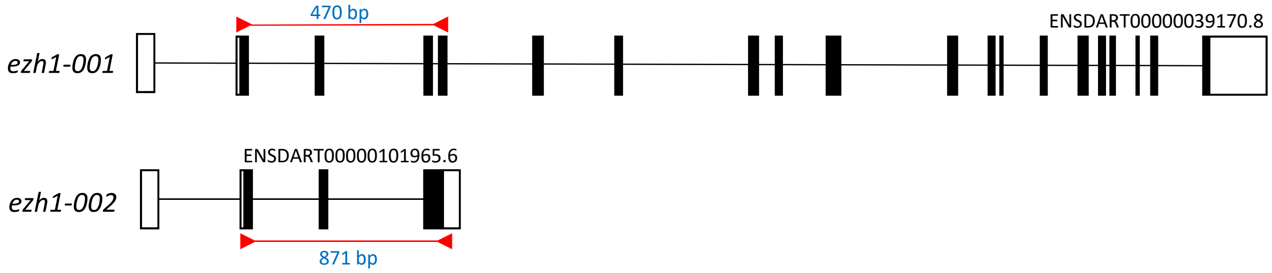

B

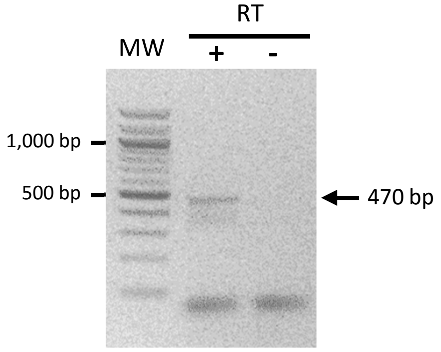

C

Danio rerio enhancer of zeste 1 polycomb repressive complex 2 subunit (ezh1), mRNA

Sequence ID: NM_001039983.2 Length: 2816 Number of Matches: 1

Range 1: 565 to 651 GenBank Graphics ▼ Next Match ▲ Previous Match

Score	Expect	Identities	Gaps	Strand
161 bits(87)	2e-38	87/87(100%)	0/87(0%)	Plus/Plus

```
Query  1    CCATTCATGTACTCCTGGTCCCCTCTGCAGCAGAACTTCATGGTGGAGGATGAGACGTTT  60
            ||||||||||||||||||||||||||||||||||||||||||||||||||||||||||||
Sbjct  565  CCATTCATGTACTCCTGGTCCCCTCTGCAGCAGAACTTCATGGTGGAGGATGAGACGTTT  624

Query  61   CTGCATAATATCCCCTACATGGGAGAT  87
            |||||||||||||||||||||||||||
Sbjct  625  CTGCATAATATCCCCTACATGGGAGAT  651
```

D

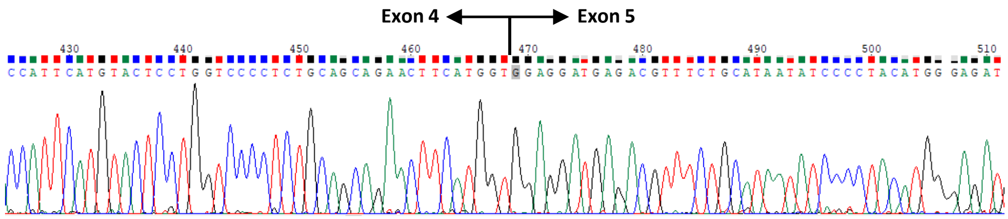

Völkel et al. – Supplementary Figure S5

A

Ezh1 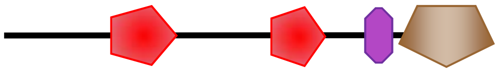 756 aa

B

>Dr_Ezh1 (NP_001035072.2)

MEEVSGPRPCTVKPRQDLLEWKKRVKSEYMRLRQLKRFRKAEQVKALFQSNRRKIEVG
TELLNEEWSKLRIQSIPLSTSSGSLPSKKLCMVEFGFPSFPNQAVAMRPLTTVAGIPF
MYSWSPLQQNFMVEDETFLHNIPYMGDEVLEQDEAFLEELIDNYDGVHGDRVSCTFLI
EGGFINDEIFKELVEALSQYSDPEEEEEEKEPTDAAENKQEEEKEMQKSAAEAPEESK
TAFFKRKWRNTAEGRELSANKKIPHDKIFTAIASMFPYKGTMQELKDKYLDLLEPSNH
VKLPPLCTPNMDGPFAKSVQREQSLHSFHTLFCRRCFKYDCFLHPFHSSPNVYKRKSK
EIHMETEPCGLDCFLLQKGAKEFADQNMMRSQRTRRRRRQPRPSSSCGHTPPDSSEKG
KEGGSDHETTSSSEGNSRCPSPIKMKPGEEESKESK**SPPQWSGAEESLFRVLHGTYYN**
NFCSIARLIGTKTCREVYEFAVKEVLIDRMPLEDSGISPQKKKRKHRLWAKIQLKKDN
SSNQVYNYQPCDHPEHPCDSSCPCVITQNFCEKFCQCDRECQ**NRFPGCRCKTQCNTKQ**
CPCYLAVRECDPDLCMTCGAADHWDSKQVSCKNCSIQRGLK**KHLLLAPSDVAGWGTFI**
KEPVQKNEFISEYCGELISQDEADRRGRIYDKYMSSFLFNLNNDFVVDATRKGNKIRF
ANHSVNPNCYAKVVMVNGDHRIGIFAKRAIQQGEELFFDYRYSQADALKYVGIEREIE
IV

C

Ezh1^{ul3} 103 aa (67 aa in common with Ezh1^{wt})

M E E V S G P R P C T V K P R Q D L L E W K K R V K S E Y M R L R Q L K
R F R K A E Q V K A L F Q S N R R K I E V G T E L L N E E W S↓S P F L C
P H P V A L C P A K S C A W W S L A F R P F R T R Q L P C V P *

Völkel et al. – Supplementary Figure S6

A

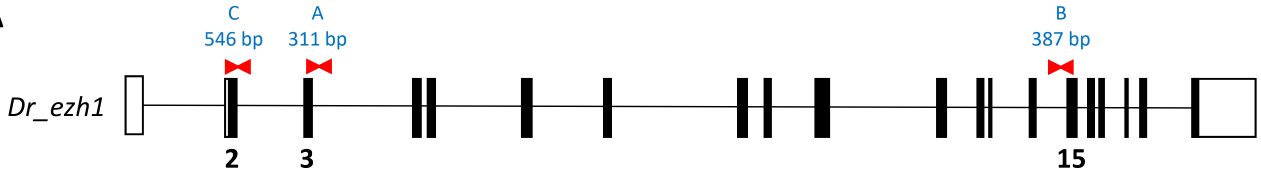

B

Primer name	Sequence	Position	Amplicon size
TAL_ezh1_5c	CCCTGCACTGTGAAGCCCAGA	C forward	546 pb
TAL_ezh1_3b	ACCACTACTGGGAAGGTGGGG	C reverse	
ezh1_5_4892fwd	GGCTCTGTTCCAGTCAAATCGCCGT	A forward	311 bp
ezh1_3_5202rev	CTGGCATGTGTTGGGGGCTCT	A reverse	
ezh1_5_27681fwd	CTGGCATGTGTTGGGGGCTCT	B forward	387 bp
ezh1_3_28067rev	TGCTGTCCCAGTGGTCTGCG	B reverse	

C

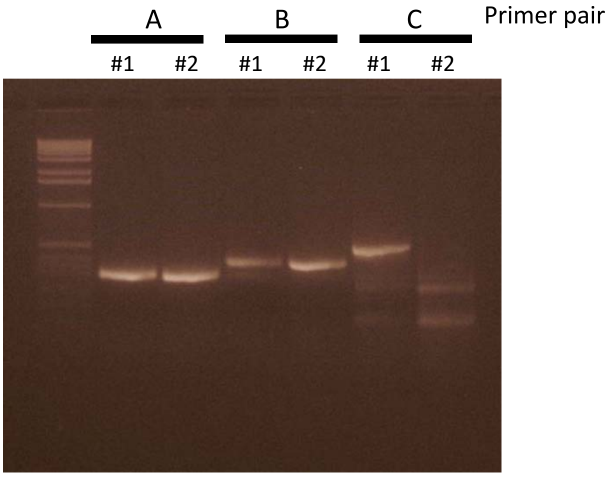

Völkel et al. – Supplementary Figure S7

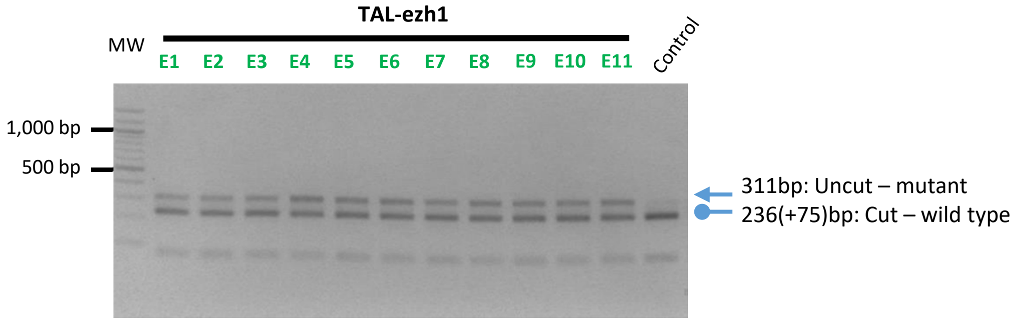

Völkel et al. – Supplementary Figure S8

Ezh1 arises from Ezh2 gene duplication but its function is not required for zebrafish development

Pamela Völkel, Aurélie Bary, Ludivine Raby, Anaïs Chapart, Barbara Dupret, Xuefen Le Bourhis and Pierre-Olivier Angrand *

ADDITIONAL INFORMATION ON RT-qPCR

This additional information on RT-qPCR includes melting curves and original RT-qPCR data used for quantification.

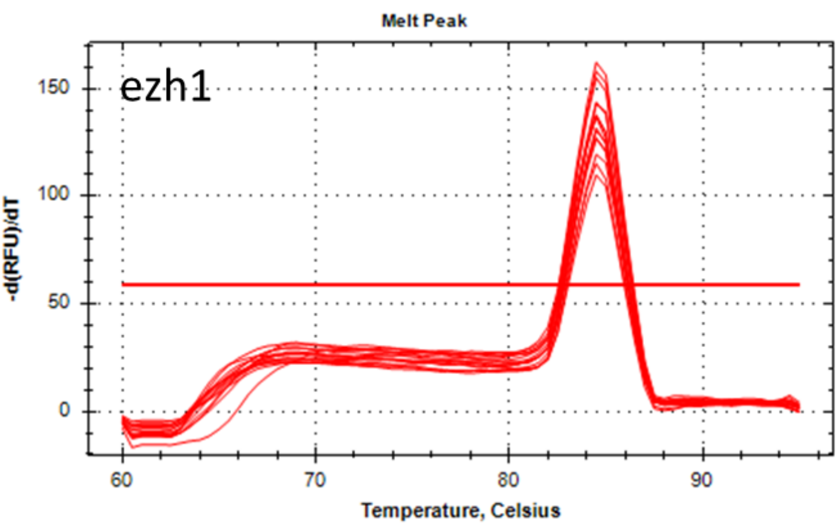
Melt Peak
ezh1
-d(RFU)/dT
150
100
50
0
60
70
80
90
Temperature, Celsius

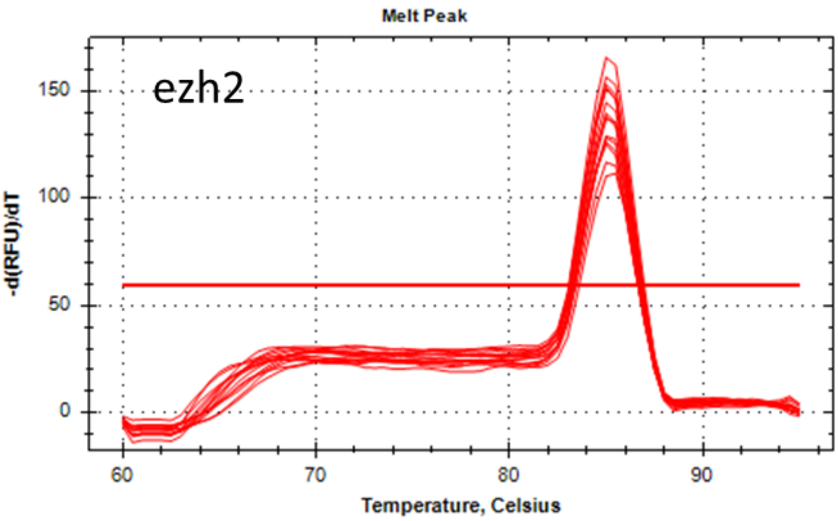
Melt Peak
ezh2
-d(RFU)/dT
150
100
50
0
60
70
80
90
Temperature, Celsius

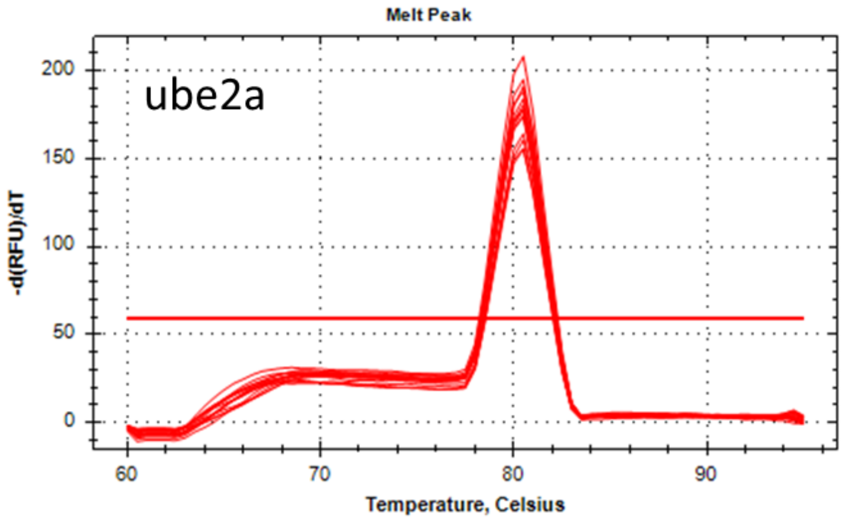
Melt Peak
ube2a
-d(RFU)/dT
200
150
100
50
0
60
70
80
90
Temperature, Celsius

File Name	qpcr 04.06.18-1.pcrd
Created By User	
Notes	
ID	
Run Started	06/04/2018 17:00:23 UTC
Run Ended	06/04/2018 18:20:48 UTC
Sample Vol	10
Lid Temp	105
Protocol File Name	Unknown.prcl
Plate Setup File Name	DefaultPlate.pltd
Base Serial Number	CT011528
Optical Head Serial Number	785BR10557
CFX Manager Version	3.1.1517.0823.

Well group	All Wells
Amplification step	3
Melt step	5

Well	Fluor	Target	Content	Sample (wt)	Cq
A01	SYBR	ube2a	Unkn	1hpf3-1	23,13
A02	SYBR	ube2a	Unkn	1hpf3-2	22,42
A03	SYBR	ube2a	Unkn	1hpf3-3	22,22
A04	SYBR	ube2a	Unkn	1hpf-RT-3	37,18
A05	SYBR	ube2a	Unkn	water	
A06	SYBR		Unkn		13,86
A07	SYBR	ezh2	Unkn	1hpf3-1	23,69
A08	SYBR	ezh2	Unkn	1hpf3-2	24,09
A09	SYBR	ezh2	Unkn	1hpf3-3	23,88
A10	SYBR	ezh2	Unkn	1hpf-RT-3	
A11	SYBR	ezh2	Unkn	water	37,28
A12	SYBR		Unkn		
B01	SYBR	ube2a	Unkn	3hpf3-1	21,35
B02	SYBR	ube2a	Unkn	3hpf3-2	20,75
B03	SYBR	ube2a	Unkn	3hpf3-3	21,11
B04	SYBR	ube2a	Unkn	3hpf-RT-3	
B05	SYBR		Unkn		
B06	SYBR		Unkn		
B07	SYBR	ezh2	Unkn	3hpf3-1	21,89
B08	SYBR	ezh2	Unkn	3hpf3-2	21,84
B09	SYBR	ezh2	Unkn	3hpf3-3	22,18
B10	SYBR	ezh2	Unkn	3hpf-RT-3	37,39
B11	SYBR		Unkn		
B12	SYBR		Unkn		

C01	SYBR	ube2a	Unkn	6hpf3-1	21,45
C02	SYBR	ube2a	Unkn	6hpf3-2	21,51
C03	SYBR	ube2a	Unkn	6hpf3-3	21,29
C04	SYBR	ube2a	Unkn	6hpf-RT-3	38,61
C05	SYBR		Unkn		
C06	SYBR		Unkn		
C07	SYBR	ezh2	Unkn	6hpf3-1	22,98
C08	SYBR	ezh2	Unkn	6hpf3-2	22,72
C09	SYBR	ezh2	Unkn	6hpf3-3	23,14
C10	SYBR	ezh2	Unkn	6hpf-RT-3	
C11	SYBR		Unkn		
C12	SYBR		Unkn		
D01	SYBR	ube2a	Unkn	1dpf3-1	21,53
D02	SYBR	ube2a	Unkn	1dpf3-2	21,00
D03	SYBR	ube2a	Unkn	1dpf3-3	20,84
D04	SYBR	ube2a	Unkn	1dpf-RT-3	
D05	SYBR		Unkn		
D06	SYBR		Unkn		
D07	SYBR	ezh2	Unkn	1dpf3-1	23,74
D08	SYBR	ezh2	Unkn	1dpf3-2	23,60
D09	SYBR	ezh2	Unkn	1dpf3-3	23,82
D10	SYBR	ezh2	Unkn	1dpf-RT-3	36,75
D11	SYBR		Unkn		
D12	SYBR		Unkn		
E01	SYBR	ube2a	Unkn	2dpf3-1	20,50
E02	SYBR	ube2a	Unkn	2dpf3-2	20,16
E03	SYBR	ube2a	Unkn	2dpf3-3	20,32
E04	SYBR	ube2a	Unkn	2dpf-RT-3	35,61
E05	SYBR		Unkn		
E06	SYBR		Unkn		
E07	SYBR	ezh2	Unkn	2dpf3-1	22,47
E08	SYBR	ezh2	Unkn	2dpf3-2	22,44
E09	SYBR	ezh2	Unkn	2dpf3-3	22,75
E10	SYBR	ezh2	Unkn	2dpf-RT-3	39,32
E11	SYBR		Unkn		
E12	SYBR		Unkn		
F01	SYBR	ube2a	Unkn	3dpf3-1	20,61
F02	SYBR	ube2a	Unkn	3dpf3-2	20,17
F03	SYBR	ube2a	Unkn	3dpf3-3	20,14
F04	SYBR	ube2a	Unkn	3dpf-RT-3	
F05	SYBR		Unkn		
F06	SYBR		Unkn		
F07	SYBR	ezh2	Unkn	3dpf3-1	23,25
F08	SYBR	ezh2	Unkn	3dpf3-2	23,20
F09	SYBR	ezh2	Unkn	3dpf3-3	23,44
F10	SYBR	ezh2	Unkn	3dpf-RT-3	37,72

F11	SYBR		Unkn		
F12	SYBR		Unkn		
G01	SYBR	ube2a	Unkn	4dpf3-1	20,76
G02	SYBR	ube2a	Unkn	4dpf3-2	20,12
G03	SYBR	ube2a	Unkn	4dpf3-3	20,35
G04	SYBR	ube2a	Unkn	4dpf-RT-3	
G05	SYBR		Unkn		
G06	SYBR		Unkn		
G07	SYBR	ezh2	Unkn	4dpf3-1	24,38
G08	SYBR	ezh2	Unkn	4dpf3-2	24,28
G09	SYBR	ezh2	Unkn	4dpf3-3	24,98
G10	SYBR	ezh2	Unkn	4dpf-RT-3	39,12
G11	SYBR		Unkn		
G12	SYBR		Unkn		
H01	SYBR	ube2a	Unkn	5dpf3-1	23,32
H02	SYBR	ube2a	Unkn	5dpf3-2	21,33
H03	SYBR	ube2a	Unkn	5dpf3-3	21,51
H04	SYBR	ube2a	Unkn	5dpf-RT-3	
H05	SYBR		Unkn		
H06	SYBR		Unkn		
H07	SYBR	ezh2	Unkn	5dpf3-1	27,38
H08	SYBR	ezh2	Unkn	5dpf3-2	27,33
H09	SYBR	ezh2	Unkn	5dpf3-3	27,60
H10	SYBR	ezh2	Unkn	5dpf-RT-3	38,27
H11	SYBR		Unkn		
H12	SYBR		Unkn		

File Name	qpcr 04.06.18-2.pcrd
Created By User	
Notes	
ID	
Run Started	06/04/2018 18:40:23 UTC
Run Ended	06/04/2018 20:00:09 UTC
Sample Vol	10
Lid Temp	105
Protocol File Name	Unknown.prcl
Plate Setup File Name	DefaultPlate.pltd
Base Serial Number	CT011528
Optical Head Serial Number	785BR10557
CFX Manager Version	3.1.1517.0823.

Well group	All Wells
Amplification step	3
Melt step	5

Well	Fluor	Target	Content	Sample (ezh1-/-)	Cq
A01	SYBR	ube2a	Unkn	1hpf3-1	21,34
A02	SYBR	ube2a	Unkn	1hpf3-2	21,26
A03	SYBR	ube2a	Unkn	1hpf3-3	21,10
A04	SYBR	ube2a	Unkn	1hpf-RT-3	
A05	SYBR	ube2a	Unkn	water	36,76
A06	SYBR		Unkn		
A07	SYBR	ezh2	Unkn	1hpf3-1	21,64
A08	SYBR	ezh2	Unkn	1hpf3-2	21,79
A09	SYBR	ezh2	Unkn	1hpf3-3	22,19
A10	SYBR	ezh2	Unkn	1hpf-RT-3	39,98
A11	SYBR	ezh2	Unkn	water	36,12
A12	SYBR		Unkn		
B01	SYBR	ube2a	Unkn	3hpf3-1	21,04
B02	SYBR	ube2a	Unkn	3hpf3-2	21,07
B03	SYBR	ube2a	Unkn	3hpf3-3	21,09
B04	SYBR	ube2a	Unkn	3hpf-RT-3	
B05	SYBR		Unkn		
B06	SYBR		Unkn		
B07	SYBR	ezh2	Unkn	3hpf3-1	20,74
B08	SYBR	ezh2	Unkn	3hpf3-2	20,81
B09	SYBR	ezh2	Unkn	3hpf3-3	20,75
B10	SYBR	ezh2	Unkn	3hpf-RT-3	35,82
B11	SYBR		Unkn		
B12	SYBR		Unkn		
C01	SYBR	ube2a	Unkn	6hpf3-1	21,35

C02	SYBR	ube2a	Unkn	6hpf3-2	21,07
C03	SYBR	ube2a	Unkn	6hpf3-3	20,77
C04	SYBR	ube2a	Unkn	6hpf-RT-3	
C05	SYBR		Unkn		
C06	SYBR		Unkn		
C07	SYBR	ezh2	Unkn	6hpf3-1	21,14
C08	SYBR	ezh2	Unkn	6hpf3-2	20,73
C09	SYBR	ezh2	Unkn	6hpf3-3	20,99
C10	SYBR	ezh2	Unkn	6hpf-RT-3	37,69
C11	SYBR		Unkn		
C12	SYBR		Unkn		
D01	SYBR	ube2a	Unkn	1dpf3-1	20,39
D02	SYBR	ube2a	Unkn	1dpf3-2	20,23
D03	SYBR	ube2a	Unkn	1dpf3-3	20,00
D04	SYBR	ube2a	Unkn	1dpf-RT-3	40,60
D05	SYBR		Unkn		
D06	SYBR		Unkn		
D07	SYBR	ezh2	Unkn	1dpf3-1	21,30
D08	SYBR	ezh2	Unkn	1dpf3-2	21,33
D09	SYBR	ezh2	Unkn	1dpf3-3	21,40
D10	SYBR	ezh2	Unkn	1dpf-RT-3	35,41
D11	SYBR		Unkn		
D12	SYBR		Unkn		
E01	SYBR	ube2a	Unkn	2dpf3-1	20,56
E02	SYBR	ube2a	Unkn	2dpf3-2	21,06
E03	SYBR	ube2a	Unkn	2dpf3-3	20,49
E04	SYBR	ube2a	Unkn	2dpf-RT-3	
E05	SYBR		Unkn		
E06	SYBR		Unkn		
E07	SYBR	ezh2	Unkn	2dpf3-1	22,26
E08	SYBR	ezh2	Unkn	2dpf3-2	22,16
E09	SYBR	ezh2	Unkn	2dpf3-3	22,50
E10	SYBR	ezh2	Unkn	2dpf-RT-3	37,04
E11	SYBR		Unkn		
E12	SYBR		Unkn		
F01	SYBR	ube2a	Unkn	3dpf3-1	20,41
F02	SYBR	ube2a	Unkn	3dpf3-2	20,17
F03	SYBR	ube2a	Unkn	3dpf3-3	20,04
F04	SYBR	ube2a	Unkn	3dpf-RT-3	
F05	SYBR		Unkn		
F06	SYBR		Unkn		
F07	SYBR	ezh2	Unkn	3dpf3-1	22,44
F08	SYBR	ezh2	Unkn	3dpf3-2	22,48
F09	SYBR	ezh2	Unkn	3dpf3-3	22,62
F10	SYBR	ezh2	Unkn	3dpf-RT-3	38,85
F11	SYBR		Unkn		

F12	SYBR		Unkn		
G01	SYBR	ube2a	Unkn	4dpf3-1	21,20
G02	SYBR	ube2a	Unkn	4dpf3-2	20,87
G03	SYBR	ube2a	Unkn	4dpf3-3	21,11
G04	SYBR	ube2a	Unkn	4dpf-RT-3	
G05	SYBR		Unkn		
G06	SYBR		Unkn		
G07	SYBR	ezh2	Unkn	4dpf3-1	24,25
G08	SYBR	ezh2	Unkn	4dpf3-2	24,46
G09	SYBR	ezh2	Unkn	4dpf3-3	24,57
G10	SYBR	ezh2	Unkn	4dpf-RT-3	36,68
G11	SYBR		Unkn		
G12	SYBR		Unkn		
H01	SYBR	ube2a	Unkn	5dpf3-1	21,34
H02	SYBR	ube2a	Unkn	5dpf3-2	21,30
H03	SYBR	ube2a	Unkn	5dpf3-3	21,08
H04	SYBR	ube2a	Unkn	5dpf-RT-3	
H05	SYBR		Unkn		
H06	SYBR		Unkn		
H07	SYBR	ezh2	Unkn	5dpf3-1	25,06
H08	SYBR	ezh2	Unkn	5dpf3-2	25,20
H09	SYBR	ezh2	Unkn	5dpf3-3	25,22
H10	SYBR	ezh2	Unkn	5dpf-RT-3	37,52
H11	SYBR		Unkn		
H12	SYBR		Unkn		

File Name	qpcr 15.11.18-A.pcrd
Created By User	
Notes	
ID	
Run Started	11/15/2018 17:53:28 UTC
Run Ended	11/15/2018 19:13:55 UTC
Sample Vol	10
Lid Temp	105
Protocol File Name	Unknown.prcl
Plate Setup File Name	DefaultPlate.pltd
Base Serial Number	CT011528
Optical Head Serial Number	785BR10557
CFX Manager Version	3.1.1517.0823.

Well group	All Wells
Amplification step	3
Melt step	5

Well	Fluor	Target	Content	Sample (wt)	Cq
A01	SYBR	ezh1	Unkn	1dpf-1-1	29,12
A02	SYBR	ezh1	Unkn	2dpf-1-1	28,96
A03	SYBR	ezh1	Unkn	3dpf-1-1	29,44
A04	SYBR	ezh1	Unkn	4dpf-1-1	27,51
A05	SYBR	ezh1	Unkn	5dpf-1-1	25,45
A06	SYBR	ezh1	Unkn	water	37,55
A07	SYBR	ezh2	Unkn	1dpf-1-1	21,50
A08	SYBR	ezh2	Unkn	2dpf-1-1	22,16
A09	SYBR	ezh2	Unkn	3dpf-1-1	24,77
A10	SYBR	ezh2	Unkn	4dpf-1-1	24,94
A11	SYBR	ezh2	Unkn	5dpf-1-1	26,12
A12	SYBR	ezh2	Unkn	water	
B01	SYBR	ezh1	Unkn	1dpf-1-2	28,82
B02	SYBR	ezh1	Unkn	2dpf-1-2	28,75
B03	SYBR	ezh1	Unkn	3dpf-1-2	29,26
B04	SYBR	ezh1	Unkn	4dpf-1-2	27,37
B05	SYBR	ezh1	Unkn	5dpf-1-2	25,10
B06	SYBR		Unkn		

B07	SYBR	ezh2	Unkn	1dpf-1-2	21,50
B08	SYBR	ezh2	Unkn	2dpf-1-2	21,76
B09	SYBR	ezh2	Unkn	3dpf-1-2	24,23
B10	SYBR	ezh2	Unkn	4dpf-1-2	24,36
B11	SYBR	ezh2	Unkn	5dpf-1-2	26,14
B12	SYBR		Unkn		
C01	SYBR	ezh1	Unkn	1dpf-1-3	28,88
C02	SYBR	ezh1	Unkn	2dpf-1-3	29,09
C03	SYBR	ezh1	Unkn	3dpf-1-3	29,34
C04	SYBR	ezh1	Unkn	4dpf-1-3	27,00
C05	SYBR	ezh1	Unkn	5dpf-1-3	25,22
C06	SYBR		Unkn		
C07	SYBR	ezh2	Unkn	1dpf-1-3	21,58
C08	SYBR	ezh2	Unkn	2dpf-1-3	21,96
C09	SYBR	ezh2	Unkn	3dpf-1-3	24,60
C10	SYBR	ezh2	Unkn	4dpf-1-3	24,18
C11	SYBR	ezh2	Unkn	5dpf-1-3	26,13
C12	SYBR		Unkn		
D01	SYBR	ezh1	Unkn	1dpf-RT-1	
D02	SYBR	ezh1	Unkn	2dpf-RT-1	
D03	SYBR	ezh1	Unkn	3dpf-RT-1	37,18
D04	SYBR	ezh1	Unkn	4dpf-RT-1	35,83
D05	SYBR	ezh1	Unkn	5dpf-RT-1	37,32
D06	SYBR		Unkn		
D07	SYBR	ezh2	Unkn	1dpf-RT-1	
D08	SYBR	ezh2	Unkn	2dpf-RT-1	
D09	SYBR	ezh2	Unkn	3dpf-RT-1	
D10	SYBR	ezh2	Unkn	4dpf-RT-1	
D11	SYBR	ezh2	Unkn	5dpf-RT-1	40,91
D12	SYBR		Unkn		
E01	SYBR	ube2a	Unkn	1dpf-1-1	20,15
E02	SYBR	ube2a	Unkn	2dpf-1-1	19,23
E03	SYBR	ube2a	Unkn	3dpf-1-1	20,26
E04	SYBR	ube2a	Unkn	4dpf-1-1	19,96
E05	SYBR	ube2a	Unkn	5dpf-1-1	20,46
E06	SYBR	ube2a	Unkn	water	
E07	SYBR		Unkn		
E08	SYBR		Unkn		

E09	SYBR		Unkn		
E10	SYBR		Unkn		
E11	SYBR		Unkn		
E12	SYBR		Unkn		
F01	SYBR	ube2a	Unkn	1dpf-1-2	20,08
F02	SYBR	ube2a	Unkn	2dpf-1-2	19,22
F03	SYBR	ube2a	Unkn	3dpf-1-2	20,15
F04	SYBR	ube2a	Unkn	4dpf-1-2	19,75
F05	SYBR	ube2a	Unkn	5dpf-1-2	21,58
F06	SYBR		Unkn		
F07	SYBR		Unkn		
F08	SYBR		Unkn		
F09	SYBR		Unkn		
F10	SYBR		Unkn		
F11	SYBR		Unkn		
F12	SYBR		Unkn		
G01	SYBR	ube2a	Unkn	1dpf-1-3	20,10
G02	SYBR	ube2a	Unkn	2dpf-1-3	19,27
G03	SYBR	ube2a	Unkn	3dpf-1-3	20,24
G04	SYBR	ube2a	Unkn	4dpf-1-3	19,82
G05	SYBR	ube2a	Unkn	5dpf-1-3	21,20
G06	SYBR		Unkn		
G07	SYBR		Unkn		
G08	SYBR		Unkn		
G09	SYBR		Unkn		
G10	SYBR		Unkn		
G11	SYBR		Unkn		
G12	SYBR		Unkn		
H01	SYBR	ube2a	Unkn	1dpf-RT-1	40,61
H02	SYBR	ube2a	Unkn	2dpf-RT-1	
H03	SYBR	ube2a	Unkn	3dpf-RT-1	
H04	SYBR	ube2a	Unkn	4dpf-RT-1	
H05	SYBR	ube2a	Unkn	5dpf-RT-1	
H06	SYBR		Unkn		
H07	SYBR		Unkn		
H08	SYBR		Unkn		
H09	SYBR		Unkn		
H10	SYBR		Unkn		

File Name	qpcr 15.11.18-B.pcrd
Created By User	
Notes	
ID	
Run Started	11/15/2018 19:28:57 UTC
Run Ended	11/15/2018 20:47:48 UTC
Sample Vol	10
Lid Temp	105
Protocol File Name	Unknown.prcl
Plate Setup File Name	DefaultPlate.pltd
Base Serial Number	CT011528
Optical Head Serial Number	785BR10557
CFX Manager Version	3.1.1517.0823.

Well group	All Wells
Amplification step	3
Melt step	5

Well	Fluor	Target	Content	Sample (wt)	Cq
A01	SYBR	ezh1	Unkn	1dpf-2-1	29,20
A02	SYBR	ezh1	Unkn	2dpf-2-1	29,01
A03	SYBR	ezh1	Unkn	3dpf-2-1	29,15
A04	SYBR	ezh1	Unkn	4dpf-2-1	27,60
A05	SYBR	ezh1	Unkn	5dpf-2-1	25,16
A06	SYBR	ezh1	Unkn	water	34,43
A07	SYBR	ezh2	Unkn	1dpf-2-1	21,22
A08	SYBR	ezh2	Unkn	2dpf-2-1	21,73
A09	SYBR	ezh2	Unkn	3dpf-2-1	24,16
A10	SYBR	ezh2	Unkn	4dpf-2-1	24,54
A11	SYBR	ezh2	Unkn	5dpf-2-1	25,74
A12	SYBR	ezh2	Unkn	water	36,38
B01	SYBR	ezh1	Unkn	1dpf-2-2	28,64
B02	SYBR	ezh1	Unkn	2dpf-2-2	28,29
B03	SYBR	ezh1	Unkn	3dpf-2-2	28,84
B04	SYBR	ezh1	Unkn	4dpf-2-2	26,21
B05	SYBR	ezh1	Unkn	5dpf-2-2	25,91
B06	SYBR		Unkn		
B07	SYBR	ezh2	Unkn	1dpf-2-2	21,55
B08	SYBR	ezh2	Unkn	2dpf-2-2	21,24
B09	SYBR	ezh2	Unkn	3dpf-2-2	24,11
B10	SYBR	ezh2	Unkn	4dpf-2-2	24,08
B11	SYBR	ezh2	Unkn	5dpf-2-2	26,50
B12	SYBR		Unkn		
C01	SYBR	ezh1	Unkn	1dpf-2-3	29,42

C02	SYBR	ezh1	Unkn	2dpf-2-3	29,67
C03	SYBR	ezh1	Unkn	3dpf-2-3	28,74
C04	SYBR	ezh1	Unkn	4dpf-2-3	27,34
C05	SYBR	ezh1	Unkn	5dpf-2-3	24,83
C06	SYBR		Unkn		
C07	SYBR	ezh2	Unkn	1dpf-2-3	21,21
C08	SYBR	ezh2	Unkn	2dpf-2-3	21,30
C09	SYBR	ezh2	Unkn	3dpf-2-3	24,10
C10	SYBR	ezh2	Unkn	4dpf-2-3	24,14
C11	SYBR	ezh2	Unkn	5dpf-2-3	25,58
C12	SYBR		Unkn		
D01	SYBR	ezh1	Unkn	1dpf-RT-2	38,20
D02	SYBR	ezh1	Unkn	2dpf-RT-2	
D03	SYBR	ezh1	Unkn	3dpf-RT-2	37,81
D04	SYBR	ezh1	Unkn	4dpf-RT-2	35,04
D05	SYBR	ezh1	Unkn	5dpf-RT-2	
D06	SYBR		Unkn		
D07	SYBR	ezh2	Unkn	1dpf-RT-2	37,15
D08	SYBR	ezh2	Unkn	2dpf-RT-2	37,05
D09	SYBR	ezh2	Unkn	3dpf-RT-2	36,43
D10	SYBR	ezh2	Unkn	4dpf-RT-2	36,45
D11	SYBR	ezh2	Unkn	5dpf-RT-2	39,98
D12	SYBR		Unkn		
E01	SYBR	ube2a	Unkn	1dpf-2-1	20,04
E02	SYBR	ube2a	Unkn	2dpf-2-1	19,43
E03	SYBR	ube2a	Unkn	3dpf-2-1	20,42
E04	SYBR	ube2a	Unkn	4dpf-2-1	19,49
E05	SYBR	ube2a	Unkn	5dpf-2-1	20,95
E06	SYBR	ube2a	Unkn	water	
E07	SYBR		Unkn		
E08	SYBR		Unkn		
E09	SYBR		Unkn		
E10	SYBR		Unkn		
E11	SYBR		Unkn		
E12	SYBR		Unkn		
F01	SYBR	ube2a	Unkn	1dpf-2-2	19,58
F02	SYBR	ube2a	Unkn	2dpf-2-2	19,29
F03	SYBR	ube2a	Unkn	3dpf-2-2	20,44
F04	SYBR	ube2a	Unkn	4dpf-2-2	19,30
F05	SYBR	ube2a	Unkn	5dpf-2-2	20,22
F06	SYBR		Unkn		
F07	SYBR		Unkn		
F08	SYBR		Unkn		
F09	SYBR		Unkn		
F10	SYBR		Unkn		
F11	SYBR		Unkn		

F12	SYBR		Unkn		
G01	SYBR	ube2a	Unkn	1dpf-2-3	20,38
G02	SYBR	ube2a	Unkn	2dpf-2-3	19,12
G03	SYBR	ube2a	Unkn	3dpf-2-3	19,90
G04	SYBR	ube2a	Unkn	4dpf-2-3	19,14
G05	SYBR	ube2a	Unkn	5dpf-2-3	21,01
G06	SYBR		Unkn		
G07	SYBR		Unkn		
G08	SYBR		Unkn		
G09	SYBR		Unkn		
G10	SYBR		Unkn		
G11	SYBR		Unkn		
G12	SYBR		Unkn		
H01	SYBR	ube2a	Unkn	1dpf-RT-2	
H02	SYBR	ube2a	Unkn	2dpf-RT-2	37,34
H03	SYBR	ube2a	Unkn	3dpf-RT-2	
H04	SYBR	ube2a	Unkn	4dpf-RT-2	
H05	SYBR	ube2a	Unkn	5dpf-RT-2	35,35
H06	SYBR		Unkn		
H07	SYBR		Unkn		
H08	SYBR		Unkn		
H09	SYBR		Unkn		
H10	SYBR		Unkn		
H11	SYBR		Unkn		
H12	SYBR		Unkn		

File Name	qpcr 15.11.18-C.pcrd
Created By User	
Notes	
ID	
Run Started	11/15/2018 21:13:26 UTC
Run Ended	11/15/2018 22:32:40 UTC
Sample Vol	10
Lid Temp	105
Protocol File Name	Unknown.prcl
Plate Setup File Name	DefaultPlate.pltd
Base Serial Number	CT011528
Optical Head Serial Number	785BR10557
CFX Manager Version	3.1.1517.0823.

Well group	All Wells
Amplification step	3
Melt step	5

Well	Fluor	Target	Content	Sample (wt)	Cq
A01	SYBR	ezh1	Unkn	1dpf-3-1	29,10
A02	SYBR	ezh1	Unkn	2dpf-3-1	29,22
A03	SYBR	ezh1	Unkn	3dpf-3-1	29,12
A04	SYBR	ezh1	Unkn	4dpf-3-1	27,60
A05	SYBR	ezh1	Unkn	5dpf-3-1	25,11
A06	SYBR	ezh1	Unkn	water	37,75
A07	SYBR	ube2a	Unkn	1dpf-3-1	19,94
A08	SYBR	ube2a	Unkn	2dpf-3-1	19,31
A09	SYBR	ube2a	Unkn	3dpf-3-1	20,11
A10	SYBR	ube2a	Unkn	4dpf-3-1	19,88
A11	SYBR	ube2a	Unkn	5dpf-3-1	21,04
A12	SYBR	ube2a	Unkn	water	
B01	SYBR	ezh1	Unkn	1dpf-3-2	28,58
B02	SYBR	ezh1	Unkn	2dpf-3-2	28,63
B03	SYBR	ezh1	Unkn	3dpf-3-2	28,53
B04	SYBR	ezh1	Unkn	4dpf-3-2	26,76
B05	SYBR	ezh1	Unkn	5dpf-3-2	25,31
B06	SYBR		Unkn		
B07	SYBR	ube2a	Unkn	1dpf-3-2	19,62
B08	SYBR	ube2a	Unkn	2dpf-3-2	19,17
B09	SYBR	ube2a	Unkn	3dpf-3-2	19,84
B10	SYBR	ube2a	Unkn	4dpf-3-2	19,56
B11	SYBR	ube2a	Unkn	5dpf-3-2	21,20
B12	SYBR		Unkn		
C01	SYBR	ezh1	Unkn	1dpf-3-3	28,56

C02	SYBR	ezh1	Unkn	2dpf-3-3	28,81
C03	SYBR	ezh1	Unkn	3dpf-3-3	28,93
C04	SYBR	ezh1	Unkn	4dpf-3-3	26,91
C05	SYBR	ezh1	Unkn	5dpf-3-3	24,99
C06	SYBR		Unkn		
C07	SYBR	ube2a	Unkn	1dpf-3-3	19,69
C08	SYBR	ube2a	Unkn	2dpf-3-3	19,08
C09	SYBR	ube2a	Unkn	3dpf-3-3	20,17
C10	SYBR	ube2a	Unkn	4dpf-3-3	19,55
C11	SYBR	ube2a	Unkn	5dpf-3-3	21,03
C12	SYBR		Unkn		
D01	SYBR	ezh1	Unkn	1dpf-RT-3	37,44
D02	SYBR	ezh1	Unkn	2dpf-RT-3	37,97
D03	SYBR	ezh1	Unkn	3dpf-RT-3	35,95
D04	SYBR	ezh1	Unkn	4dpf-RT-3	38,53
D05	SYBR	ezh1	Unkn	5dpf-RT-3	36,97
D06	SYBR		Unkn		
D07	SYBR	ube2a	Unkn	1dpf-RT-3	
D08	SYBR	ube2a	Unkn	2dpf-RT-3	37,44
D09	SYBR	ube2a	Unkn	3dpf-RT-3	
D10	SYBR	ube2a	Unkn	4dpf-RT-3	
D11	SYBR	ube2a	Unkn	5dpf-RT-3	
D12	SYBR		Unkn		
E01	SYBR		Unkn		
E02	SYBR		Unkn		
E03	SYBR		Unkn		
E04	SYBR		Unkn		
E05	SYBR		Unkn		
E06	SYBR		Unkn		
E07	SYBR		Unkn		
E08	SYBR		Unkn		
E09	SYBR		Unkn		
E10	SYBR		Unkn		
E11	SYBR		Unkn		
E12	SYBR		Unkn		
F01	SYBR		Unkn		
F02	SYBR		Unkn		
F03	SYBR		Unkn		
F04	SYBR		Unkn		
F05	SYBR		Unkn		
F06	SYBR		Unkn		
F07	SYBR		Unkn		
F08	SYBR		Unkn		
F09	SYBR		Unkn		
F10	SYBR		Unkn		
F11	SYBR		Unkn		

File Name	qpcr 15.11.18-D.pcrd
Created By User	
Notes	
ID	
Run Started	11/15/2018 22:55:10 UTC
Run Ended	11/16/2018 00:14:17 UTC
Sample Vol	10
Lid Temp	105
Protocol File Name	Unknown.prcl
Plate Setup File Name	DefaultPlate.pltd
Base Serial Number	CT011528
Optical Head Serial Number	785BR10557
CFX Manager Version	3.1.1517.0823.

Well group	All Wells
Amplification step	3
Melt step	5

Well	Fluor	Target	Content	Sample (ezh1-/-)	Cq
A01	SYBR	ezh2	Unkn	1dpf-1-1	21,12
A02	SYBR	ezh2	Unkn	2dpf-1-1	22,03
A03	SYBR	ezh2	Unkn	3dpf-1-1	21,71
A04	SYBR	ezh2	Unkn	4dpf-1-1	23,70
A05	SYBR	ezh2	Unkn	5dpf-1-1	24,19
A06	SYBR	ezh2	Unkn	water	34,41
A07	SYBR	ezh2	Unkn	1dpf-2-1	20,80
A08	SYBR	ezh2	Unkn	2dpf-2-1	21,65
A09	SYBR	ezh2	Unkn	3dpf-2-1	22,32
A10	SYBR	ezh2	Unkn	4dpf-2-1	23,42
A11	SYBR	ezh2	Unkn	5dpf-2-1	24,75
A12	SYBR	ezh2	Unkn	water	35,51
B01	SYBR	ezh2	Unkn	1dpf-1-2	21,14
B02	SYBR	ezh2	Unkn	2dpf-1-2	21,13
B03	SYBR	ezh2	Unkn	3dpf-1-2	22,13
B04	SYBR	ezh2	Unkn	4dpf-1-2	23,48
B05	SYBR	ezh2	Unkn	5dpf-1-2	24,41
B06	SYBR		Unkn		
B07	SYBR	ezh2	Unkn	1dpf-2-2	20,74
B08	SYBR	ezh2	Unkn	2dpf-2-2	21,64
B09	SYBR	ezh2	Unkn	3dpf-2-2	21,80
B10	SYBR	ezh2	Unkn	4dpf-2-2	23,15
B11	SYBR	ezh2	Unkn	5dpf-2-2	24,00
B12	SYBR		Unkn		
C01	SYBR	ezh2	Unkn	1dpf-1-3	21,02
C02	SYBR	ezh2	Unkn	2dpf-1-3	21,56

C03	SYBR	ezh2	Unkn	3dpf-1-3	22,00
C04	SYBR	ezh2	Unkn	4dpf-1-3	23,54
C05	SYBR	ezh2	Unkn	5dpf-1-3	24,25
C06	SYBR		Unkn		
C07	SYBR	ezh2	Unkn	1dpf-2-3	20,94
C08	SYBR	ezh2	Unkn	2dpf-2-3	21,38
C09	SYBR	ezh2	Unkn	3dpf-2-3	22,06
C10	SYBR	ezh2	Unkn	4dpf-2-3	23,02
C11	SYBR	ezh2	Unkn	5dpf-2-3	24,56
C12	SYBR		Unkn		
D01	SYBR	ezh2	Unkn	1dpf-RT-1	34,73
D02	SYBR	ezh2	Unkn	2dpf-RT-1	35,48
D03	SYBR	ezh2	Unkn	3dpf-RT-1	34,86
D04	SYBR	ezh2	Unkn	4dpf-RT-1	35,32
D05	SYBR	ezh2	Unkn	5dpf-RT-1	34,71
D06	SYBR		Unkn		
D07	SYBR	ezh2	Unkn	1dpf-RT-2	34,31
D08	SYBR	ezh2	Unkn	2dpf-RT-2	34,77
D09	SYBR	ezh2	Unkn	3dpf-RT-2	34,64
D10	SYBR	ezh2	Unkn	4dpf-RT-2	35,16
D11	SYBR	ezh2	Unkn	5dpf-RT-2	35,54
D12	SYBR		Unkn		
E01	SYBR	ube2a	Unkn	1dpf-1-1	20,06
E02	SYBR	ube2a	Unkn	2dpf-1-1	19,52
E03	SYBR	ube2a	Unkn	3dpf-1-1	18,93
E04	SYBR	ube2a	Unkn	4dpf-1-1	20,21
E05	SYBR	ube2a	Unkn	5dpf-1-1	19,56
E06	SYBR	ube2a	Unkn	water	
E07	SYBR	ube2a	Unkn	1dpf-2-1	19,31
E08	SYBR	ube2a	Unkn	2dpf-2-1	19,34
E09	SYBR	ube2a	Unkn	3dpf-2-1	19,24
E10	SYBR	ube2a	Unkn	4dpf-2-1	20,21
E11	SYBR	ube2a	Unkn	5dpf-2-1	20,13
E12	SYBR	ube2a	Unkn	water	
F01	SYBR	ube2a	Unkn	1dpf-1-2	19,51
F02	SYBR	ube2a	Unkn	2dpf-1-2	19,81
F03	SYBR	ube2a	Unkn	3dpf-1-2	19,23
F04	SYBR	ube2a	Unkn	4dpf-1-2	20,16
F05	SYBR	ube2a	Unkn	5dpf-1-2	19,81
F06	SYBR		Unkn		
F07	SYBR	ube2a	Unkn	1dpf-2-2	19,06
F08	SYBR	ube2a	Unkn	2dpf-2-2	19,27
F09	SYBR	ube2a	Unkn	3dpf-2-2	19,30
F10	SYBR	ube2a	Unkn	4dpf-2-2	19,84
F11	SYBR	ube2a	Unkn	5dpf-2-2	20,17
F12	SYBR		Unkn		

G01	SYBR	ube2a	Unkn	1dpf-1-3	20,10
G02	SYBR	ube2a	Unkn	2dpf-1-3	19,67
G03	SYBR	ube2a	Unkn	3dpf-1-3	19,65
G04	SYBR	ube2a	Unkn	4dpf-1-3	19,63
G05	SYBR	ube2a	Unkn	5dpf-1-3	20,29
G06	SYBR		Unkn		
G07	SYBR	ube2a	Unkn	1dpf-2-3	19,05
G08	SYBR	ube2a	Unkn	2dpf-2-3	19,61
G09	SYBR	ube2a	Unkn	3dpf-2-3	19,29
G10	SYBR	ube2a	Unkn	4dpf-2-3	20,01
G11	SYBR	ube2a	Unkn	5dpf-2-3	20,09
G12	SYBR		Unkn		
H01	SYBR	ube2a	Unkn	1dpf-RT-1	
H02	SYBR	ube2a	Unkn	2dpf-RT-1	38,02
H03	SYBR	ube2a	Unkn	3dpf-RT-1	
H04	SYBR	ube2a	Unkn	4dpf-RT-1	
H05	SYBR	ube2a	Unkn	5dpf-RT-1	
H06	SYBR		Unkn		
H07	SYBR	ube2a	Unkn	1dpf-RT-2	
H08	SYBR	ube2a	Unkn	2dpf-RT-2	
H09	SYBR	ube2a	Unkn	3dpf-RT-2	
H10	SYBR	ube2a	Unkn	4dpf-RT-2	
H11	SYBR	ube2a	Unkn	5dpf-RT-2	
H12	SYBR		Unkn		
